# Bridging Machine Learning and Algorithmic Information Theory, Part VII: Algorithmic Information Kernels and Kernel Discrepancies on Countable Spaces

**DOI:** 10.3390/e28101084

**Published:** 2026-10-01

**Authors:** Boumediene Hamzi, Marcus Hutter, Houman Owhadi

**Affiliations:** 1 Department of Computing and Mathematical Sciences, California Institute of Technology, Pasadena, CA 91125, USA; 2 The Alan Turing Institute, London NW1 2DB, UK; 3 Google DeepMind, London N1C 4DJ, UK; 4 School of Computing, Australian National University, Canberra, ACT 2601, Australia

**Keywords:** Kolmogorov complexity, Solomonoff induction, algorithmic mutual information, normalized compression distance, reproducing kernel Hilbert space, maximum mean discrepancy, Hilbert–Schmidt independence criterion, kernel Stein discrepancy, characteristic kernels, hypothesis testing, 68Q30, 46E22, 62G10, 60J10

## Abstract

Compression-based dissimilarities such as the normalized compression distance are widely used, but direct exponentiation need not produce a positive semidefinite kernel. We develop a systematic interface between prefix algorithmic information theory and three kernel discrepancy methods on finite or countable spaces: maximum mean discrepancy (MMD), the Hilbert–Schmidt independence criterion (HSIC), and a Markov-operator kernel Stein discrepancy (KSD). Explicit feature realizations guarantee positive semidefiniteness for distance-to-kernel embeddings and for an ideal Solomonoff feature-mixture kernel satisfying $k_{\mathrm{Sol},U}\left(x,y\right){≍}_{U}2^{-K_{U}\left(\langle x,y\rangle \right)}$. Zero-mass signed measures give the exact characteristicity criterion, and whether the ideal Solomonoff kernel meets that criterion turns out to depend on the universal machine: two universal prefix machines are exhibited, one whose kernel is not characteristic on a two-point set and one whose kernel separates all finite-total-variation signed measures on the countable string space. In the resulting RKHS geometry, the MMD compares compressibility profiles, the HSIC is product-kernel MMD from the joint law to the product of its marginals, and ${\mathrm{KSD}}_{T_{P},k}\left(Q\right)={\mathrm{MMD}}_{k}\left(QT_{P},Q\right)$ for a target-stationary Markov kernel $T_{P}$. Expected algorithmic mutual information is connected to the HSIC only through explicit source-description, mutual-information, support, and kernel conditions. Ideal AIT objects, computable feature surrogates, and finite-sample errors are separated explicitly. Reproducible synthetic experiments examine the identities, landmark approximation, and selected testing alternatives at the computable and empirical layers. They provide no evidence about ideal AIT quantities or general superiority over baselines. An algebraic example also shows that a characteristic finite-support kernel can give a constant permutation statistic: identification alone does not guarantee test power.

## 1. Introduction

Algorithmic information theory measures the complexity of an individual object by the length of its shortest program. Kernel statistics measures differences between probability distributions by inner products in a reproducing-kernel Hilbert space. The first is absolute and uncomputable; the second is statistical, tractable, and equipped with finite-sample theory. This paper asks what a rigorous interface between them looks like: which algorithmic constructions are valid positive semidefinite kernels, what the standard discrepancies mean once those kernels are used, and what the exact countable-space analogue of the kernel Stein discrepancy is.

The interface runs in two directions, and they are not symmetric. Going from algorithmic information to kernels, one obtains unconditional constructions: compression dissimilarities become kernels, and the resulting geometry can be characterized exactly. Going from kernels back to algorithmic information, one obtains interpretations and one-sided bounds whose converses require explicit extra hypotheses.

**Figure 1:**
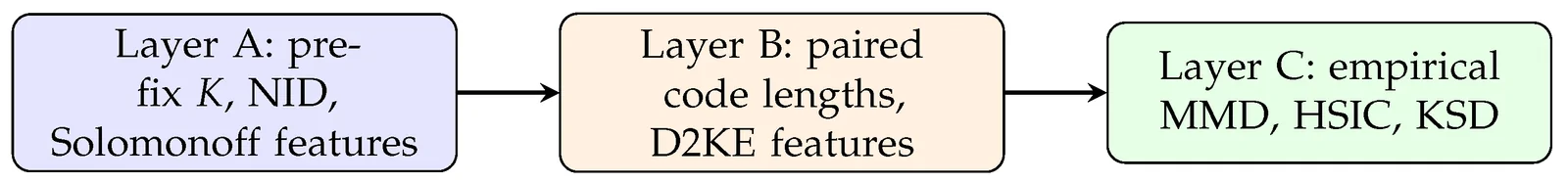
The three layers: blue denotes Layer A, orange Layer B, and green Layer C. Arrows carry explicit hypotheses and error bounds; no general guarantee is asserted for any particular off-the-shelf compressor.

Cutting across both directions is a three-layer discipline, shown in Figure 1. Layer A contains ideal objects defined from a fixed universal prefix machine. Layer B contains computable, compressor-relative features. Layer C contains empirical statistics computed from finitely many samples.

The link between them is clear. A kernel k(x,y) is a measure of similarity: it is large when *x* and *y* are alike. Conditional Kolmogorov complexity measures the same thing with the opposite orientation, since K(x∣y) is small when *y* already supplies most of what is needed to produce *x*, and large when it supplies nothing. This observation is where the present series began: Part II [1] introduced Kolmogorov-complexity kernels (KC-kernels) built from dissimilarities such as NCD, conditional complexity, and algorithmic mutual information. One way to connect the two notions is to exponentiate a negative, as in $k\left(x,y\right)=e^{-\gamma \;\mathrm{NCD}(x,y)}$.

That particular formula fails: the resulting matrix need not be positive semidefinite. The distance-to-kernel embedding of [2] repairs this by introducing landmarks. In D2KE, a landmark is an ordinary reference object from a chosen landmark space Λ; in the present string setting, it is typically another string. Landmarks are not class labels or cluster centres, and they need not be among the particular observations *x* and *y* being compared. D2KE does not prescribe one unique landmark set: choosing the landmarks is part of specifying the kernel. Mathematically, one chooses a landmark distribution π on Λ, which determines which reference objects are used and how much weight they receive; the population kernel averages over this distribution. In a finite computation, one may draw *m* landmarks from π, use an external reference corpus or a held-out data split, or select a representative subset of the pooled observations without using their labels. For a goodness-of-fit problem, landmarks may instead be drawn from the target model. The aim is to cover the kinds of patterns and scales that the data may contain, while keeping the choice independent of the labels or test outcome. Once chosen, the exact same landmarks and weights are used for every input and every pairwise comparison. Each landmark asks one simple question—how similar is this input to that reference object?—and the answers provide a common coordinate system. For practical KC-kernels, one chooses a compressor-based dissimilarity $d_{C}$—for example, NCD or a proxy based on conditional code length—and a finite sample of landmarks $\omega_{1},\ldots ,\omega_{m}$. The input *x* is then represented by the list of similarities $e^{-\gamma d_{C}\left(x,\omega_{j}\right)}$ to those landmarks, and the kernel between *x* and *y* is the inner product of their two lists, $k_{C}^{\left(m\right)}\left(x,y\right)=m^{-1}\sum_{j=1}^{m}e^{-\gamma d_{C}\left(x,\omega_{j}\right)}e^{-\gamma d_{C}\left(y,\omega_{j}\right)}$. Thus, two objects are similar when they have similar relationships to the same reference strings; the construction is positive semidefinite because it is an inner product, regardless of whether $d_{C}$ itself has the properties of a kernel distance. The Solomonoff kernel uses the same landmark principle at the ideal AIT level, but replaces the finite, compressor-dependent landmark set by the infinite set Ω of all halting prefix programs. A program ω acts as a possible shared explanation; its coordinate for *x* is $2^{-K(x|\omega )}$, and shorter programs receive the larger universal-prior weight $2^{-|\omega |}$. Hence $k_{\mathrm{Sol}}\left(x,y\right)=\sum_{\omega \in \Omega }2^{-|\omega |}2^{-K(x|\omega )}2^{-K(y|\omega )}$, so two strings score highly when the same short programs explain both. In short, the D2KE KC-kernels of Part III [3] are finite, computable, and depend on a chosen compressor and landmark sample, whereas the Solomonoff kernels and feature mixtures of Part IV [4] use all halting programs and exact Kolmogorov complexities, making them canonical ideal constructions but uncomputable in practice.

Once the geometry is fixed, each discrepancy acquires a reading. The MMD becomes the $L^{2}$ distance between compressibility profiles, so the two laws are indistinguishable exactly when almost every landmark under π observes the same average compression from both. The HSIC becomes an MMD with a product kernel between the joint law and the product of its marginals, so it vanishes when the joint compressibility profile factors. The Stein discrepancy becomes an MMD between a law and its own one-step image under a model-stationary Markov kernel, so it vanishes when one step of the chosen target-stationary operator leaves the candidate law unchanged. This auxiliary operator need not be the physical transition law that produced the observations; stationary-law agreement does not identify transition dynamics. The delicate connection is the one running from expected algorithmic mutual information through Shannon mutual information and Pinsker’s inequality to the HSIC: it says that objects sharing little algorithmic information on average will also look independent to a kernel test, and its converse holds only under support and eigenvalue conditions we state explicitly.

- In the AIT-to-kernel direction, we recall the Solomonoff feature kernel and pair-mass equivalence from Part IV [4] (Proposition 7), giving a self-contained semimeasure proof under explicit program-domain assumptions. We derive its normalized form and study the machine dependence of characteristicity. We specialize the kernel mean-embedding criterion to zero-mass signed measures in these feature families, with an explicit finite-support rank certification.
- In the kernel-to-AIT direction, we prove that the MMD under these kernels is exactly the $L^{2}$ distance between landmark compressibility profiles; we combine the product-kernel reading of the HSIC with the classical expected-AMI theorem for computable sources to obtain a one-sided AMI–HSIC bound, and a two-sided comparison whose constant we identify as the product of the restricted minimum eigenvalues of the two marginal Gram matrices; and we establish the exact discrete Stein identity ${\mathrm{KSD}}_{T_{P},k}\left(Q\right)={\mathrm{MMD}}_{k}\left(QT_{P},Q\right)$, together with its quadratic Stein kernel and zero-set conditions.

Throughout, the passage from ideal objects to computable surrogates and then to finite samples carries explicit constants. The MMD and HSIC randomization tests have finite-sample level control under the exchangeability and feature-selection conditions of Proposition 15. The KSD goodness-of-fit procedure uses the separate exact-target simulation conditions in Remark 29.

The components we draw on are individually standard. The MMD, HSIC, and KSD are established tools in nonparametric statistics and machine learning. The MMD provides a reproducing-kernel characterization of differences between probability laws and underpins a large literature on two-sample testing and distribution comparison; the classical framework and finite-sample testing consequences were established by [5].

The HSIC is a kernel dependence measure obtained from the Hilbert–Schmidt norm of a cross-covariance operator; it vanishes under independence and, under characteristic-kernel assumptions, detects independence. The foundational formulation is due to [6].

The KSD extends Stein’s method into reproducing-kernel form and yields goodness-of-fit procedures that compare a candidate law with data through a Stein operator and a kernelized discrepancy. Central references include [7,8]; extensions to discrete spaces have been studied by [9].

A complementary line of work has established deep ties between distance-based and RKHS-based testing statistics. This literature includes distance covariance for Euclidean random vectors [10] and its extension to metric spaces [11]. The authors of [12] showed an equivalence between distance-based and RKHS-based statistics in hypothesis testing, clarifying when semimetrics of negative type and reproducing kernels represent the same inferential content.

The theory of characteristic kernels and kernel mean embeddings was developed by [13,14,15]. A kernel is characteristic if the map from distributions to their mean embeddings is injective; this property ensures population identification against every distinct law in the specified class. It is not necessary for power against a particular alternative and does not by itself guarantee finite-sample power.

On the algorithmic-information side, the relevant foundations are due to [16,17,18,19,20,21]. Information distance and normalized variants were developed by [22,23]. The normalized compression distance (NCD) was introduced by [24] as a practical approximation. A comprehensive treatment appears in the monograph by [25].

The use of compression for similarity measurement has a long history; see [23] for an overview. The challenge of obtaining valid positive semidefinite kernels from compression-based dissimilarities was addressed by the D2KE construction of [2], applied to compression dissimilarities in [26].

This paper is Part VII of an open-ended series [1,3,4,26,27,28] that systematically develops the interface between AIT and kernel-based ML. Part I introduced complexity-based kernel learning; Part II extended the programme to unsupervised learning; and Part III developed the spectral and learning-rate theory and applied the D2KE construction of [2] to compression dissimilarities. Parts IV–VI added the probabilistic layer: Solomonoff Gaussian processes and Solomonoff Gaussian Hilbert Spaces (Part IV), learning theory in SGHS and numerical validation (Part V), and extensions to deep architectures (Part VI). The present paper is self-contained: every definition and every hypothesis it uses is restated here, and the earlier parts are cited as ordinary references rather than as prerequisites. A broader synthesis is given in the research monograph preprint by Hamzi and Hutter [29]. The present paper adds the discrepancy layer: it does not reprove the classical theory of the MMD, HSIC, or KSD, nor resolve the uncomputability of Kolmogorov complexity. Rather, it isolates mathematically clean interfaces between kernel discrepancies and algorithmic information: (i) turning compression dissimilarities into PSD kernels, with an exact characteristicity criterion, (ii) interpreting discrepancy measures through compressibility profiles, (iii) connecting the HSIC to expected algorithmic mutual information under explicitly stated source, support, and kernel conditions, and (iv) clarifying which statements survive when one moves from ideal AIT objects to practical compressor surrogates.

Recent work has explored using compressors directly for classification via a “gzip classifier” [30], which attracted significant attention and debate. Our framework provides a PSD kernelization route for related compressor dissimilarities: D2KE makes the Gram matrix valid by construction. It does not, by itself, supply a classification guarantee or identify a compressor length with Kolmogorov complexity.

The relationship between stochastic properties of data and Kolmogorov complexity has been studied in the algorithmic statistics literature [31]. The notion of “algorithmic sufficient statistics” provides a complementary perspective to our compressibility profiles (Definition 22).

The question of whether a kernel is “universal” (with RKHS dense in C(X)) is related to but distinct from characteristicity [32]. Gaussian kernels are universal; whether D2KE kernels with NCD dissimilarities inherit any universality property is an open question connected to the characteristicity questions in Section 8.18.

### 1.1. What the Existing Approaches Give, and What They Do Not

The three ingredients below are individually mature, and each leaves a specific gap that this paper is written to close.

Information distance and compression distances require different qualifications. Ideal information distance is defined directly from prefix complexity and has universality properties under the stated admissibility conditions [22,23]. Practical NCD applies a chosen compressor and serialization to raw objects without requiring handcrafted vector features [24]. It therefore depends on those choices, and neither a generic approximation to prefix complexity nor positive semidefiniteness of $e^{-\gamma {\mathrm{NCD}}_{C}}$ follows automatically. A valid kernel construction is needed for the RKHS discrepancy framework used here.

Kernel discrepancies supply exact or asymptotically exact tests, finite-sample bounds, and a clean detection theory under characteristic kernels [5,6,7,8]. What they presuppose is a valid kernel, which may itself be defined on structured objects. This theory does not select a unique compressor-relative geometry, and its identification conditions must still be checked for the selected feature family. A countable-space Stein construction also requires a selected target-stationary operator.

The distance-to-kernel embedding of [2] removes the first obstruction: any non-negative dissimilarity becomes a PSD kernel through landmark features, with computable random-feature approximations. That work discusses the choice of landmark distribution, including representative-set and synthetic choices for strings, time series, and sets; it also proves RKHS Lipschitz properties under metric assumptions and analyzes approximation and generalization error. The additional question here is distributional identification and the interpretation of the MMD, HSIC, and Markov KSD for compression and program-feature kernels. Earlier parts of this series applied D2KE to compression dissimilarities and developed the spectral, learning-rate, and probabilistic layers [3,4]; the discrepancy layer was not treated, and characteristicity was handled through sufficient conditions.

Table 1 states the resulting division of labour. The contribution of this paper is not a new discrepancy or a new compressor but the interface: an exact mathematical identification criterion and a finite-support matrix certification, an interpretation of each discrepancy for the selected feature family, and explicit hypotheses for passing between ideal objects, compressors, and finite samples. The criterion is not an algorithm that decides characteristicity for arbitrary kernels or universal machines.

### 1.2. Practical Significance

The framework specifies two-sample and independence procedures on raw byte strings, using compressor-derived features and permutation calibration under Proposition 15. The goodness-of-fit procedure instead uses exact target simulation when it is available. The restricted minimum Gram eigenvalue $\alpha_{k}$ certifies identification on a specified finite support; it does not guarantee power. These operational statements and the limited scope of the synthetic experiments are distinguished in Section 8.17.

Several limits on what follows should be stated at the outset; the scope of the numerical evidence in particular is set out in Section 8.17. The ideal Solomonoff kernel remains uncomputable. Finite-support characteristicity requires the exact affine zero-mass condition or a stronger rank/margin condition; distinct strings alone do not imply the latter. Practical compressor guarantees are conditional on a stated code-length approximation hypothesis. The numerical study of Section 8 is confined to Layers B and C and makes no claim about any Layer-A quantity.

**Figure 2:**
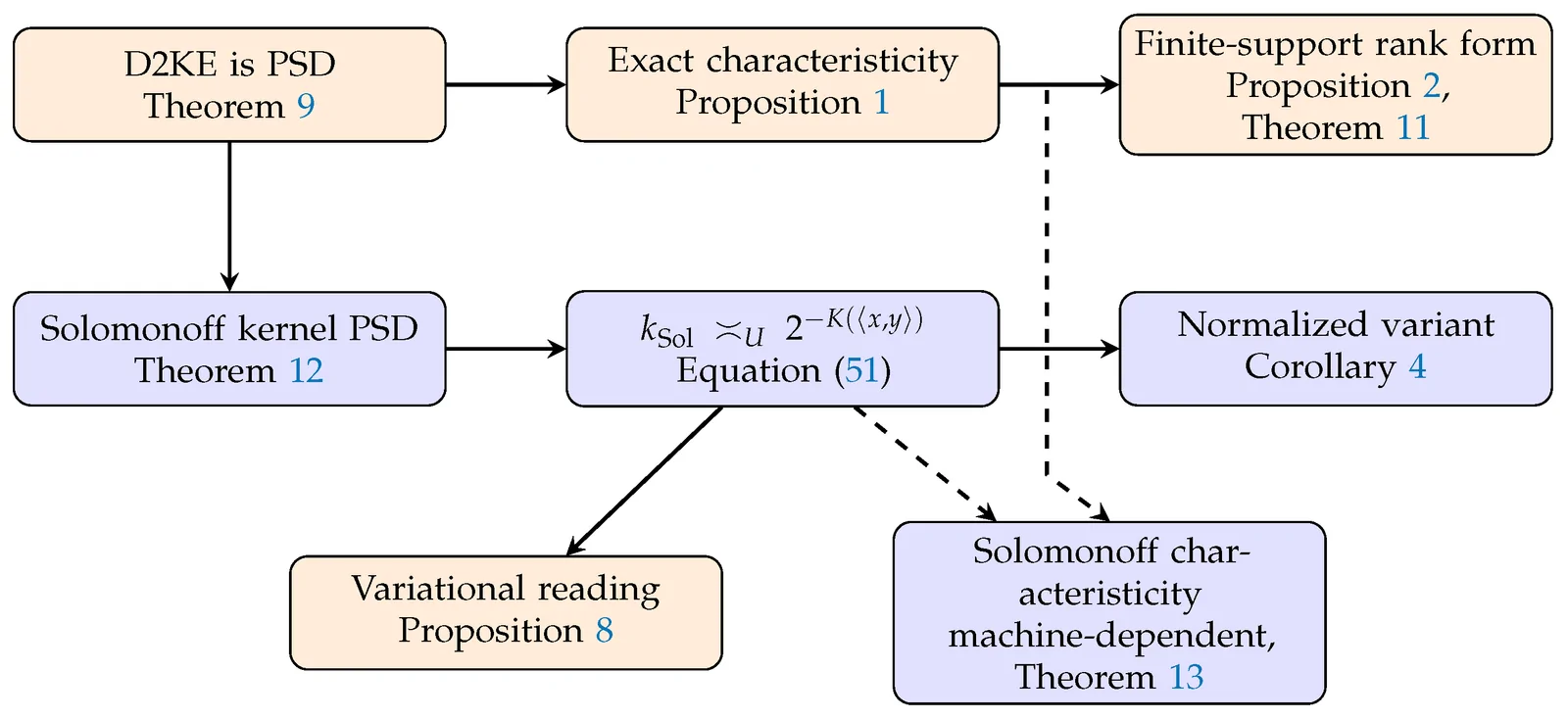
Direction I, from algorithmic information to kernels (Section 4). Blue is Layer A, orange Layer B. Solid arrows are logical dependence, dashed arrows conceptual motivation.

**Figure 3:**
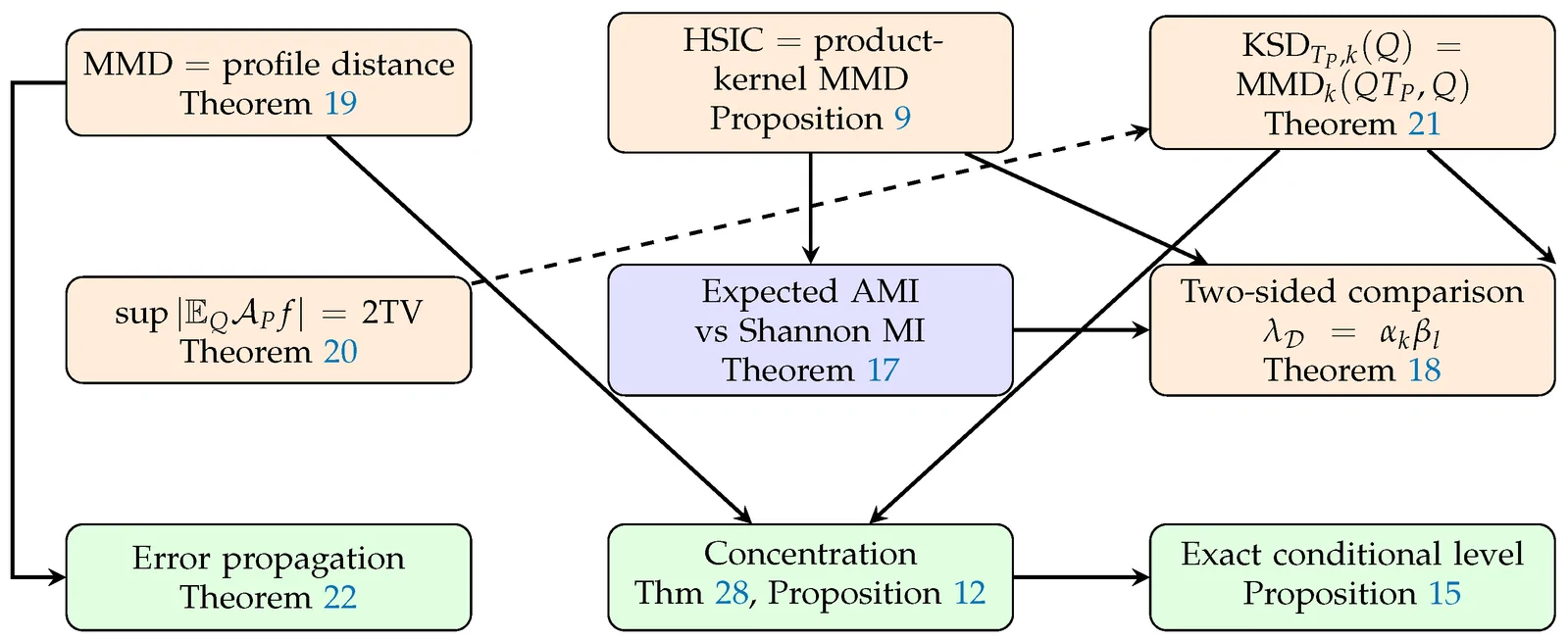
Direction II, from kernel discrepancies back to algorithmic information (Section 5), and the passage to finite samples (Section 6 and Section 7). Blue denotes Layer A, orange Layer B, and green Layer C.

The paper is organized as follows. Section 2 and Section 3 fix notation and recall what is needed from each side. Section 4 develops the first direction, from algorithmic information to kernels; Section 5 develops the second, from kernel discrepancies back to algorithmic quantities. Section 6 isolates the computability gap and propagates approximation error through both directions, and Section 7 adds the sampling error. Section 8 collects the resulting protocols, the numerical evidence, the limitations, and the open problems. Figure 2 and Figure 3 map the dependencies among the main results and may be read as a summary of the whole paper.

## 2. Background I: Algorithmic Information

This section and the next fix the two vocabularies the paper joins. Nothing here is new; the purpose is to make the hypotheses of Section 4 and Section 5 checkable without recourse to other sources, and to pin down the pairing and serialization conventions that later cause trouble if left implicit. Everything in this section lives at Layer A of Figure 1.

This section provides a self-contained introduction to the AIT concepts used in this paper. Readers familiar with Kolmogorov complexity may skim this section; those new to the area should read it carefully.

### 2.1. Kolmogorov Complexity

**Definition** **1** (Universal prefix machine)**.** *A partial computable machine U(p,z) with auxiliary input z is a* universal prefix machine *if, for every z, its halting program domain is prefix-free and it simulates every other prefix machine with an additive program-length constant. We fix one such machine throughout and write U(p):=U(p,ϵ) when no auxiliary input is supplied.*

**Definition** **2** (Kolmogorov Complexity)**.** *For the fixed universal prefix machine U, the* prefix Kolmogorov complexity *of a finite binary string $x\in {\left\{0,1\right\}}^{*}$ is*

(1) K(x)=min{|p|:U(p)=x};

*the length of the shortest program that outputs x and halts.*

**Theorem** **1** (Invariance Theorem [16])**.** *For any two universal Turing machines $U_{1}$ and $U_{2}$, there exists a constant c (depending only on $U_{1}$ and $U_{2}$) such that for all $x\in {\left\{0,1\right\}}^{*}$:*

(2) $$|K_{U_{1}}\left(x\right)-K_{U_{2}}\left(x\right)|\leq c.$$

This theorem justifies suppressing the dependence on the choice of universal machine, as it affects complexity only by an additive constant.

**Definition** **3** (Conditional Complexity)**.** *The* conditional Kolmogorov complexity *of x given y is*

(3) K(x|y)=min{|p|:U(p,y)=x},

*where U(p,y) denotes the output of the same prefix machine on program p with auxiliary input y. Prefix-freeness is in the program argument p, uniformly in y.*

**Definition** **4** (Joint Complexity)**.** *The* joint Kolmogorov complexity *of x and y is*

(4) K(x,y)=K(〈x,y〉),

*where 〈x,y〉 is a standard pairing function (e.g., prefix-free encoding of the pair).*

**Remark** **1** (Notation: Pairing vs. Concatenation)**.** *Throughout the paper, K(x,y) denotes the complexity of a fixed self-delimiting pair 〈x,y〉. Compressor joint lengths use the separate, explicitly specified serialization ${\langle x,y\rangle }_{C}$ and its symmetrized length $\ell_{C}^{\mathrm{pair}}\left(x,y\right)$. We do not use ambiguous bare concatenation in a definition or algorithm.*

### 2.2. Fundamental Inequalities

**Theorem** **2** (Symmetry of Information (Kolmogorov–Levin) [20,25,33])**.** *For all $x,y\in {\left\{0,1\right\}}^{*}$:*

(5) $$K\left(x,y\right)=K\left(x\right)+K\left(y|x^{*}\right)+O\left(\log K\left(x,y\right)\right)=K\left(y\right)+K\left(x|y^{*}\right)+O\left(\log K\left(x,y\right)\right),$$

*where $x^{*}$ denotes the shortest program for x.*

**Proof reference.**  The lower bound is a nontrivial counting result: a shortest program cannot in general be computed from its output. We therefore invoke the standard Kolmogorov–Levin symmetry-of-information theorem; see [25] (Chapter 3). No computability of $x^{*}$ from (x,y) is assumed.    □

**Corollary** **1** (Chain Rule)**.** *K(x,y)=K(x)+K(y|x)+O(logK(x,y)).*

**Proof.**  From the shortest program $x^{*}$, one computes *x* (so $K\left(x|x^{*}\right)=O\left(1\right)$). Conversely, from (x,K(x)), one can dovetail all programs of length K(x) until one is found that outputs *x*; a self-delimiting description of the integer K(x) costs O(logK(x)) bits. Thus, $K\left(x^{*}|x\right)=O\left(\log K\left(x\right)\right)$ for the canonically selected shortest program, and $K\left(y|x\right)=K\left(y|x^{*}\right)+O\left(\log K\left(x\right)\right)$; substituting into Theorem 2 absorbs the difference into the O(logK(x,y)) error term.    □

### 2.3. Algorithmic Mutual Information

**Definition** **5** (Algorithmic Mutual Information)**.** *The* algorithmic mutual information *between x and y is*

(6) AMI(x;y)=K(x)+K(y)−K(x,y).

By the symmetry of information (Theorem 2):

(7) $$\mathrm{AMI}\left(x;y\right)=K\left(x\right)-K\left(x|y^{*}\right)+O\left(\log K\left(x,y\right)\right)=K\left(y\right)-K\left(y|x^{*}\right)+O\left(\log K\left(x,y\right)\right).$$

**Remark** **2.** *Unlike Shannon mutual information, the AMI is defined for individual objects rather than random variables. It measures the amount of information that knowing one object provides about the other, in terms of program length.*

### 2.4. Information Distance and NID

**Definition** **6** (Information Distance [22])**.** *The* information distance *between x and y is*

(8) E(x,y)=max{K(x|y),K(y|x)}.

**Theorem** **3** ([22])**.** *E(x,y) is a metric on ${\left\{0,1\right\}}^{*}$ up to O(log) terms.*

**Definition** **7** (Normalized Information Distance)**.** *The* normalized information distance *is*

(9) $$\mathrm{NID}\left(x,y\right)=\frac{\max\{K(x|y),K(y|x)\}}{\max\{K(x),K(y)\}}.$$

**Theorem** **4** ([23])**.** *Up to normalized lower-order additive terms, the NID takes values in [0,1] and satisfies the metric axioms. Its universality statement holds over the admissible upper semicomputable normalized distances satisfying the density condition of [23]; it is not a statement about every computable function called a distance.*

### 2.5. Normalized Compression Distance

Since K(·) is uncomputable, practical applications use compressor-based approximations.

**Definition** **8** (Normalized Compression Distance)**.** *For a compressor C with code-length function $\ell_{C}$, let ${\langle x,y\rangle }_{C}$ be an explicitly self-delimiting serialization of the ordered pair and define the symmetric paired length*

(10) $$\ell_{C}^{\mathrm{pair}}\left(x,y\right):=\min\left\{\ell_{C}\left({\langle x,y\rangle }_{C}\right),\ell_{C}\left({\langle y,x\rangle }_{C}\right)\right\}.$$

*Then,*

(11) $${\mathrm{NCD}}_{C}\left(x,y\right)=\frac{\ell_{C}^{\mathrm{pair}}\left(x,y\right)-\min\left\{\ell_{C}\left(x\right),\ell_{C}\left(y\right)\right\}}{\max\{\ell_{C}\left(x\right),\ell_{C}\left(y\right)\}},$$

*with the paired length defined above.*

**Remark** **3.** *For an ideal code satisfying $\ell_{C}\left(x\right)=K\left(x\right)+O\left(1\right)$ on the relevant paired objects, ${\mathrm{NCD}}_{C}$ approximates the NID up to the symmetry-of-information and serialization overheads made explicit in Theorem 22. For real compressors (gzip, bzip2, LZMA), no such approximation guarantee is asserted here; it would require a separate compressor- and source-specific theorem.*

### 2.6. Solomonoff Semimeasure

**Definition** **9** (Solomonoff Semimeasure)**.** *The* Solomonoff semimeasure *(or universal prior) is*

(12) $$M\left(x\right)=\sum_{p:U(p)=x}2^{-|p|};$$

*the probability that a uniformly random infinite binary string, when fed as a program to U, produces x as output.*

**Theorem** **5** (Coding Theorem [20])**.**

(13) $$-\log_{2}M\left(x\right)=K\left(x\right)+O\left(1\right).$$

This theorem connects the probabilistic (Solomonoff) and algorithmic (Kolmogorov) viewpoints: the negative log-probability under the universal prior equals the Kolmogorov complexity up to a constant.

## 3. Background II: Kernel Discrepancies on Countable Spaces

Where Section 2 supplied absolute, uncomputable quantities attached to individual objects, this section supplies relative, estimable quantities attached to distributions. We restrict throughout to finite or countable domains, which is the setting in which strings live and in which the exact criteria of Section 4 hold. The one departure from textbook treatment is characteristicity, which we state as an exact criterion on zero-mass signed measures rather than as a list of sufficient conditions, because that is the form the later results need.

This section provides a self-contained introduction to kernel methods and discrepancy measures.

### 3.1. Reproducing Kernel Hilbert Spaces

Standard background on kernels and RKHSs is given by Steinwart and Christmann [34].

**Definition** **10** (Positive Semidefinite Kernel)**.** *A function k:X×X→R is a* positive semidefinite (PSD) kernel *if it is symmetric, k(x,y)=k(y,x), and, for all n≥1, all $x_{1},\ldots ,x_{n}\in X$, and all $c_{1},\ldots ,c_{n}\in R$,*

(14) $$\sum_{i=1}^{n}\sum_{j=1}^{n}c_{i}c_{j}k\left(x_{i},x_{j}\right)\geq 0.$$

**Theorem** **6** (Moore–Aronszajn)**.** *For every PSD kernel k, there exists a unique Hilbert space $H_{k}$ of functions f:X→R such that:*

(i) $k\left(\cdot ,x\right)\in H_{k}$* for all *x∈X*.*
(ii) $f\left(x\right)={\langle f,k\left(\cdot ,x\right)\rangle }_{H_{k}}$* for all *$f\in H_{k}$* (reproducing property).*

$H_{k}$* is called the* reproducing kernel Hilbert space *(RKHS) of k.*

**Definition** **11** (Feature Map)**.** *A* feature map *for kernel k is a map ϕ:X→H (for some Hilbert space H) such that $k\left(x,y\right)={\langle \phi \left(x\right),\phi \left(y\right)\rangle }_{H}$.*

### 3.2. Kernel Mean Embeddings

**Definition** **12** (Kernel Mean Embedding)**.** *For a probability measure P on X and a bounded kernel k, the* kernel mean embedding *of P is*

(15) $$\mu_{P}=E_{X∼P}\left[k\left(\cdot ,X\right)\right]\in H_{k}.$$

The key property is:

(16) $$E_{X∼P}\left[f\left(X\right)\right]={\langle f,\mu_{P}\rangle }_{H_{k}}\;\mathrm{for}\;\mathrm{all}\;f\in H_{k}.$$

**Definition** **13** (Characteristic Kernel)**.** *A kernel k is* characteristic *(for a class P of probability measures) if the map $P\mapsto \mu_{P}$ is injective on P. Equivalently, $\mu_{P}=\mu_{Q}$ implies that P=Q for P,Q∈P.*

For the remainder of the paper, X is finite or countably infinite unless a result explicitly states otherwise. Write

(17) $$M_{0}\left(X\right):=\left\{\nu :\sum_{x\in X}\left|\nu \left(x\right)\right|<\infty ,\;\sum_{x\in X}\nu \left(x\right)=0\right\}.$$

For a bounded PSD kernel, define

(18) $$\mu_{k}\left(\nu \right):=\sum_{x}\nu \left(x\right)k\left(x,\cdot \right),\;E_{k}\left(\nu \right):={∥\mu_{k}\left(\nu \right)∥}_{H_{k}}^{2}=\sum_{x,y}k\left(x,y\right)\nu \left(x\right)\nu \left(y\right).$$

**Proposition** **1** (Zero-mass signed-measure criterion)**.** *A bounded PSD kernel on a countable space is characteristic on all probability measures if and only if*

(19) $$E_{k}\left(\nu \right)>0\;\mathrm{for}\;\mathrm{every}\;\mathrm{nonzero}\;\nu \in M_{0}\left(X\right).$$

**Proof.**  If P≠Q, then P−Q is a nonzero element of $M_{0}\left(X\right)$ and $E_{k}\left(P-Q\right)={∥\mu_{P}-\mu_{Q}∥}_{H_{k}}^{2}$. Conversely, for nonzero $\nu \in M_{0}\left(X\right)$, its Jordan parts have the same positive mass *a*. Thus, $P=\nu^{+}/a$ and $Q=\nu^{-}/a$ are distinct probabilities and $\mu_{k}\left(\nu \right)=a\left(\mu_{P}-\mu_{Q}\right)$. This proves both directions.    □

**Definition** **14** (Characteristic on Finite Support)**.** *Let P(S) denote probability measures supported on a finite set S⊂X. A kernel k is* characteristic on *Sif $P\mapsto \mu_{P}$ is injective on P(S).*

**Proposition** **2** (Finite-state matrix criterion)**.** *Let $S=\{x_{1},\ldots ,x_{N}\}$ and let $K_{S}={\left(k\left(x_{i},x_{j}\right)\right)}_{i,j}$. The kernel is characteristic on P(S) if and only if*

(20) $$a^{⊤}K_{S}a>0\;\mathrm{for}\;\mathrm{every}\;a\neq 0\;\mathrm{satisfying}\;1^{⊤}a=0.$$

*Equivalently, its feature vectors are affinely independent. Strict positive definiteness is sufficient but not necessary.*

**Proof.**  On *S*, a zero-mass signed measure is exactly a coefficient vector *a* with $1^{⊤}a=0$, and its energy is $a^{⊤}K_{S}a$. Apply Proposition 1.    □

**Example** **1** (Point separation is insufficient)**.** *On S={1,2,3}, let ϕ(1)=0, ϕ(2)=1 and ϕ(3)=2. The scalar feature is injective, but *

(21) $$\mu_{k}\left(\delta_{2}\right)=1=\mu_{k}\;\left(\frac{1}{2}\delta_{1}+\frac{1}{2}\delta_{3}\right).$$

*Hence, injectivity on points does not imply distributional identification.*

### 3.3. Maximum Mean Discrepancy

**Definition** **15** (MMD)**.** *The* maximum mean discrepancy *between distributions P and Q is*

(22) $${\mathrm{MMD}}_{k}\left(P,Q\right)={∥\mu_{P}-\mu_{Q}∥}_{H_{k}}.$$

**Remark** **4** (Total-variation convention, used throughout)**.** *For a signed measure ν, we write ${∥\nu ∥}_{\mathrm{TV}}$ for its total mass (the $L^{1}$ norm) and $\mathrm{TV}\left(\mu ,\nu \right):=\frac{1}{2}{∥\mu -\nu ∥}_{\mathrm{TV}}$ for the half-normalized (probabilists’) distance, so that 0≤TV(μ,ν)≤1 for probability measures and ${∥\mu -\nu ∥}_{\mathrm{TV}}=2\;\mathrm{TV}\left(\mu ,\nu \right)$. This convention is used in Theorem 17, Theorem 20, and Proposition 11.*

**Proposition** **3** ([5])**.**

(23) $${\mathrm{MMD}}_{k}^{2}\left(P,Q\right)=E_{X,X^{\prime }∼P}\left[k\left(X,X^{\prime }\right)\right]-2E_{X∼P,Y∼Q}\left[k\left(X,Y\right)\right]+E_{Y,Y^{\prime }∼Q}\left[k\left(Y,Y^{\prime }\right)\right].$$

**Theorem** **7** ([5])**.** *If k is characteristic, then ${\mathrm{MMD}}_{k}$ is a metric on probability measures: ${\mathrm{MMD}}_{k}\left(P,Q\right)=0$ if and only if P=Q.*

### 3.4. Hilbert–Schmidt Independence Criterion

**Definition** **16** (Cross-Covariance Operator)**.** *For joint distribution $P_{XY}$ on X×Y with kernels k on X and l on Y, the* cross-covariance operator *$C_{XY}:H_{l}\to H_{k}$ is defined by*

(24) $$C_{XY}=E_{\left(X,Y\right)∼P_{XY}}\left[k\left(\cdot ,X\right)⊗l\left(\cdot ,Y\right)\right]-E_{X∼P_{X}}\left[k\left(\cdot ,X\right)\right]⊗E_{Y∼P_{Y}}\left[l\left(\cdot ,Y\right)\right].$$

**Definition** **17** (HSIC)**.** *The* Hilbert–Schmidt independence criterion *is*

(25) $$\mathrm{HSIC}\left(P_{XY};k,l\right)={∥C_{XY}∥}_{\mathrm{HS}}^{2},$$

*the squared Hilbert–Schmidt norm of the cross-covariance operator.*

**Theorem** **8** ([6,35])**.** *Suppose the product kernel k⊗l is independence-characteristic for the relevant class. In particular, this class contains $P_{XY}$ and $P_{X}⊗P_{Y}$. Then, $\mathrm{HSIC}(P_{XY};k,l)=0$ if and only if X⊥Y. On finite supports, characteristic marginal kernels are sufficient.*

### 3.5. Stein Discrepancies and Kernel Stein Discrepancy

Let *P* be a target law and let $A_{P}$ be a linear operator satisfying the Stein identity $E_{P}\left[A_{P}f\right]=0$ on a test-function class F. The corresponding Stein discrepancy is

(26) $$S_{P,F}\left(Q\right):=\underset{f\in F}{\sup}\left|E_{Q}\left[A_{P}f\right]\right|.$$

A kernel Stein discrepancy chooses F from an RKHS unit ball, or an operator-transformed RKHS unit ball, so that the supremum becomes the norm of an expected Stein feature and admits a quadratic kernel representation [7,8,36]. Classical continuous KSDs use score-based differential Stein operators. On a countable space there is no canonical differential operator, so Section 5.5 uses the target-stationary Markov operator $A_{P}=T_{P}-I$. It proves directly that the resulting KSD is exactly ${\mathrm{MMD}}_{k}\left(QT_{P},Q\right)$, and states the additional conditions under which its zero set is {P}.

## 4. Direction I: From Algorithmic Information to Kernels

The first of the two directions announced in Section 1 runs from algorithmic information to kernels. It is the stronger half of the interface: the constructions here are unconditional, and the geometry they produce can be characterized exactly using the criterion of Section 3. We begin with the obstruction that makes the naive construction fail, resolve it with the D2KE landmark embedding of [2], determine precisely when the resulting kernel separates distributions, and then push the same idea to its ideal limit by taking the halting programs of Section 2 as landmarks. Figure 2 maps the dependencies.

This section develops the first direction of our bridge: using AIT concepts to construct principled kernels.

### 4.1. The PSD Problem

The most direct transcription of that intuition into a kernel is

(27) $$k_{\mathrm{NCD}}\left(x,y\right)=\exp\left(-\gamma \;{\mathrm{NCD}}_{C}\left(x,y\right)\right),\;\gamma >0,$$

and Part II [1] develops this together with the related KC-kernels built from conditional complexity and algorithmic mutual information. It needs nothing beyond a compressor, which is the appeal, and it carries no guarantee of being a kernel. By Schoenberg’s theorem, $e^{-\gamma d}$ is positive semidefinite for every γ>0 exactly when the symmetrized *d* is conditionally of negative type; nothing in the definition of ${\mathrm{NCD}}_{C}$ supplies that property, and for a real, order-sensitive compressor it must not be assumed. Every algorithm below therefore uses D2KE, or an explicitly verified PSD alternative, rather than the raw plug-in expression. The evidence here is one-sided: an empirical Gram matrix can disprove positive semidefiniteness for a particular compressor and dataset, but no amount of computation establishes it for all NCD implementations.

### 4.2. The Distance-to-Kernel Embedding (D2KE)

The distance-to-kernel embedding of [2] resolves the problem by a change of perspective, and earlier parts of this series applied it to compression dissimilarities [3,26].

**Definition** **18** (D2KE kernel, [2])**.** *Let (X,A) be a measurable space, (Λ,F,π) a finite positive measure space, and d:X×Λ→[0,∞) a measurable non-negative dissimilarity. For bandwidth γ>0, define the feature map $\phi :X\to L^{2}\left(\Lambda ,\pi \right)$ by*

(28) $$\phi \left(x\right)\left(\omega \right)=e^{-\gamma d(x,\omega )}.$$

*The* D2KE kernel *is*

(29) $$k_{D2\mathrm{KE}}\left(x,y\right)={\langle \phi \left(x\right),\phi \left(y\right)\rangle }_{L^{2}\left(\pi \right)}=\int_{\Lambda }e^{-\gamma d(x,\omega )}e^{-\gamma d(y,\omega )}\;d\pi \left(\omega \right).$$

**Remark** **5** (Notation: landmark space versus program space)**.** *Throughout,* Λ *denotes the* landmark *space of the D2KE construction and π its landmark measure. The symbol* Ω *is reserved for the prefix-free halting domain dom(U) of the universal machine in the Solomonoff kernel of Section 4, where the measure $\omega \mapsto 2^{-|\omega |}$ is a subprobability. The two spaces play formally analogous roles but belong to different layers:* Λ *is chosen by the user and is computable,* Ω *is not. Gram matrices are written $G_{k},G_{l}$ so that K is reserved for prefix complexity.*

The key idea is that instead of measuring similarity between *x* and *y* directly (which may fail to be PSD), we measure how each object relates to a shared collection of landmarks ω, and then compare these profiles. Because the kernel is an average of products of non-negative features, the resulting Gram matrix is always a sum of rank-one PSD terms—just like a sample covariance matrix.

**Theorem** **9** (D2KE is always PSD)**.** *The kernel $k_{D2\mathrm{KE}}$ defined in (29) is positive semidefinite on X. Moreover,*

(30) $${\mathrm{MMD}}_{k_{D2\mathrm{KE}}}^{2}\left(P,Q\right)={∥E_{X∼P}\left[\phi \left(X\right)\right]-E_{Y∼Q}\left[\phi \left(Y\right)\right]∥}_{L^{2}\left(\pi \right)}^{2}.$$

**Proof.**  Choose arbitrary $x_{1},\ldots ,x_{n}\in X$ and $c_{1},\ldots ,c_{n}\in R$. We must show that

(31) $$\sum_{i,j}c_{i}c_{j}k_{D2\mathrm{KE}}\left(x_{i},x_{j}\right)\geq 0.$$

**Step 1: Expand the sum using the definition.**

(32) $$\begin{array}{cc} \sum_{i=1}^{n}\sum_{j=1}^{n}c_{i}c_{j}k_{D2\mathrm{KE}}\left(x_{i},x_{j}\right)\; & =\sum_{i=1}^{n}\sum_{j=1}^{n}c_{i}c_{j}\int_{\Lambda }e^{-\gamma d(x_{i},\omega )}e^{-\gamma d(x_{j},\omega )}\;d\pi \left(\omega \right). \end{array}$$

**Step 2: Exchange sum and integral.** Since the sum is finite and the integrand is bounded (each factor is in (0,1]), we can exchange the order by Fubini’s theorem:

(33) $$\begin{array}{cc} \; & =\int_{\Lambda }\sum_{i=1}^{n}\sum_{j=1}^{n}c_{i}c_{j}e^{-\gamma d(x_{i},\omega )}e^{-\gamma d(x_{j},\omega )}\;d\pi \left(\omega \right). \end{array}$$

**Step 3: Recognize a perfect square.**

(34) $$\begin{array}{cc} \; & =\int_{\Lambda }\left(\sum_{i=1}^{n}c_{i}e^{-\gamma d(x_{i},\omega )}\right)\left(\sum_{j=1}^{n}c_{j}e^{-\gamma d(x_{j},\omega )}\right)d\pi \left(\omega \right), \end{array}$$

(35) $$\begin{array}{cc} \; & =\int_{\Lambda }{\left(\sum_{i=1}^{n}c_{i}e^{-\gamma d(x_{i},\omega )}\right)}^{2}d\pi \left(\omega \right). \end{array}$$

**Step 4: Conclude non-negativity.** The integrand is a perfect square, hence non-negative pointwise. Integrating a non-negative function against a positive measure yields a non-negative result. Therefore,

(36) $$\sum_{i,j}c_{i}c_{j}k_{D2\mathrm{KE}}\left(x_{i},x_{j}\right)\geq 0.$$

**Step 5: MMD identity.** Since $k_{D2\mathrm{KE}}\left(x,y\right)={\langle \phi \left(x\right),\phi \left(y\right)\rangle }_{L^{2}\left(\pi \right)}$, the RKHS $H_{k_{D2\mathrm{KE}}}$ is isometrically isomorphic to the closure of span{ϕ(x):x∈X} in $L^{2}\left(\pi \right)$. The kernel mean embedding is:

(37) $$\mu_{P}=E_{X∼P}\left[\phi \left(X\right)\right]\in L^{2}\left(\pi \right).$$

Therefore,

(38) $${\mathrm{MMD}}_{k_{D2\mathrm{KE}}}^{2}\left(P,Q\right)={∥\mu_{P}-\mu_{Q}∥}_{L^{2}\left(\pi \right)}^{2}={∥E_{X∼P}\left[\phi \left(X\right)\right]-E_{Y∼Q}\left[\phi \left(Y\right)\right]∥}_{L^{2}\left(\pi \right)}^{2}.\;□$$

### 4.3. Characteristicity of D2KE Kernels

**Definition** **19** ($L^{2}\left(\pi \right)$-determining landmarks)**.** *The landmark family $\left\{e^{-\gamma d(\cdot ,\omega )}:\omega \in \Lambda \right\}$ is *$L^{2}\left(\pi \right)$-determining *for a class P of probability measures on X if, for all distinct P,Q∈P,*

(39) $$\pi \;\left(\left\{\omega :E_{X∼P}e^{-\gamma d(X,\omega )}\neq E_{Y∼Q}e^{-\gamma d(Y,\omega )}\right\}\right)>0.$$

**Theorem** **10** (Exact characteristicity criterion for D2KE)**.** *The kernel $k_{D2\mathrm{KE}}$ is characteristic on a class P if and only if the landmark family is $L^{2}\left(\pi \right)$-determining for P in the sense of Definition 19. In particular, it is characteristic on all probability measures exactly when every nonzero $\nu \in M_{0}\left(X\right)$ satisfies*

(40) $$\pi \;\left(\left\{\omega :\sum_{x}\nu \left(x\right)e^{-\gamma d(x,\omega )}\neq 0\right\}\right)>0.$$

**Proof.**  The kernel mean embedding of *P* is the function $\mu_{P}\left(\omega \right)=E_{X∼P}\left[e^{-\gamma d(X,\omega )}\right]$. If $\mu_{P}=\mu_{Q}$ in $L^{2}\left(\pi \right)$, their profiles agree π-almost everywhere. The determining condition states that this is impossible for P≠Q; hence, P=Q. Conversely, if the profiles differ on a set of positive π-measure, their squared $L^{2}\left(\pi \right)$ distance is positive. The signed-measure form follows from Proposition 1 and the identity

(41) $$E_{k_{D2\mathrm{KE}}}\left(\nu \right)=\int_{\Lambda }{\left(\sum_{x}\nu \left(x\right)e^{-\gamma d(x,\omega )}\right)}^{2}d\pi \left(\omega \right).$$

   □

**Remark** **6** (Point separation is *not* sufficient)**.** *Pointwise separation—e.g., a single $\omega_{0}$ with $d\left(x,\omega_{0}\right)\neq d\left(y,\omega_{0}\right)$ for all x≠y, making one feature injective—is* not sufficient *for the determining property: distinct measures P≠Q can share the same single-feature mean $\mu_{P}\left(\omega_{0}\right)=\mu_{Q}\left(\omega_{0}\right)$, so an injective scalar feature does not determine the measure. A determining family requires the features to be total (a dense/characteristic family), not merely point-separating.*

The following theorem provides a clean, checkable criterion for characteristicity on finite supports that does not require the separating property to hold globally.

**Theorem** **11** (Finite-Support Characteristicity for D2KE)**.** *Let $X=\{x_{1},\ldots ,x_{n}\}$ be a finite set and suppose that $\omega_{1},\ldots ,\omega_{m}$ are atoms with $\pi (\left\{\omega_{j}\right\})>0$. Define*

(42) $$A={\left(A_{ij}\right)}_{1\leq i\leq n,\;1\leq j\leq m},\;A_{ij}:=e^{-\gamma d(x_{i},\omega_{j})}.$$

*Then, $k_{D2\mathrm{KE}}$ is characteristic on all probability measures on X whenever*

(43) $$aA=0\;\mathrm{and}\;\sum_{i=1}^{n}a_{i}=0\;⟹\;a=0.$$

*This affine-independence condition is also necessary when the selected atoms exhaust the landmark support. A full row rank of A is a stronger sufficient condition. For a kernel using exactly these m landmark features, n>m+1 forces failure of affine independence; such a kernel can still distinguish particular alternatives.*

**Proof.**  Let *P* and *Q* be probability measures on X. Write their mass vectors as $p={\left(p_{i}\right)}_{i=1}^{n}$ and $q={\left(q_{i}\right)}_{i=1}^{n}$. Their kernel mean embeddings are

(44) $$\mu_{P}=\sum_{i}p_{i}\phi \left(x_{i}\right),\;\mu_{Q}=\sum_{i}q_{i}\phi \left(x_{i}\right).$$

Suppose $\mu_{P}=\mu_{Q}$ in $L^{2}\left(\pi \right)$. Then, $\sum_{i=1}^{n}\left(p_{i}-q_{i}\right)\phi \left(x_{i}\right)=0$. Evaluating at each $\omega_{j}$ yields

(45) $$\sum_{i=1}^{n}\left(p_{i}-q_{i}\right)e^{-\gamma d(x_{i},\omega_{j})}=0,\;j=1,\ldots ,m,$$

which is (p−q)A=0. Since *p* and *q* are probability vectors, $\sum_{i}\left(p_{i}-q_{i}\right)=0$. Condition (43) therefore gives p=q. Conversely, if the selected atoms exhaust the landmark support and a nonzero zero-sum vector *a* satisfies aA=0, then sufficiently small positive and negative perturbations of an interior probability vector in the direction *a* produce distinct probability laws with the same mean embedding.    □

**Corollary** **2.** *If m=n and the square matrix $A_{ij}=e^{-\gamma d(x_{i},\omega_{j})}$ is invertible, then $k_{D2\mathrm{KE}}$ is characteristic on all probability measures on X.*

**Remark** **7.** *Theorem 11 applies to every D2KE kernel. For the Solomonoff feature family, unconditional characteristicity on every finite set of distinct strings is not inferred from point separation or from the pair-complexity equivalence proved below; it holds for some universal machines and fails for others (Theorem 13).*

### 4.4. The Solomonoff Kernel

We now introduce an ideal kernel defined using prefix-free algorithmic information. The intuition is as follows: two strings *x* and *y* should be “similar” if there exists a short program ω that explains both well (i.e., K(x|ω) and K(y|ω) are both small). The Solomonoff kernel formalizes this by summing over *all* halting programs ω, weighted by the universal prior $2^{-|\omega |}$, with each program contributing a term that decays exponentially in how hard it is to reconstruct *x* and *y* from ω. This is “similarity through shared algorithmic explanation.”

**Definition** **20** (Solomonoff Kernel)**.** *Fix a universal prefix Turing machine U, and let*

(46) Ω:=dom(U)

*be its prefix-free domain of halting programs. Here, U is the same universal* conditional *prefix machine that supplies the conditional complexities $K\left(x|\omega \right)=K_{U}\left(x|\omega \right)$ below, and dom(U) is read through the convention U(p)=U(p,ϵ) fixed in Background I, so that Ω=dom(U(·,ϵ)). Program set and conditional complexities are therefore read off the same machine, which is what makes the constructions of Propositions 5 and 6 well posed. Define*

(47) $$\pi \left(\omega \right):=2^{-|\omega |},\;\omega \in \Omega ,$$

*so that π is the canonical finite subprobability measure on the prefix-free program set* Ω; *by Kraft’s inequality $Z:=\sum_{\omega \in \Omega }2^{-|\omega |}\in \left(0,1\right]$, and we leave π unnormalized. The* Solomonoff kernel *is*

(48) $$k_{\mathrm{Sol}}\left(x,y\right):=\sum_{\omega \in \Omega }2^{-|\omega |}\cdot 2^{-K(x|\omega )}\cdot 2^{-K(y|\omega )}.$$

**Remark** **8** (Notation)**.** *Throughout the Solomonoff-kernel construction, the symbol ω denotes a halting prefix program belonging to the prefix-free domain Ω=dom(U) of the fixed universal prefix machine U. We reserve other symbols, such as $z\in {\left\{0,1\right\}}^{*}$, for arbitrary finite binary side information when needed outside the kernel definition itself.*

**Remark** **9** (Ideal object vs. practical workhorse)**.** *The Solomonoff kernel is an* ideal object: *it depends on prefix Kolmogorov complexity and on a universal prefix machine, and is therefore not computable. Its role in this paper is conceptual and structural: it provides a mathematically natural AIT kernel that serves as the conceptual “North Star” for the theory—a target object rather than one for which a general approximation theorem from compressor-based kernels is supplied (see Section 8.2). For all implementable algorithms, the D2KE construction with real-world compressors (Section 4.2 and Section 6) is the practical workhorse, realizing a separate, compressor-relative population kernel. The Solomonoff kernel sets the* conceptual *target; no general theorem certifies that D2KE with compressors converges to it (Section 8.2).*

The pair-mass equivalence below was stated in Hamzi et al. [4] (Proposition 7). We give a self-contained proof using lower-semicomputable semimeasure domination and a shortest pair program, with the program domain and conditional-prefix conventions fixed in Definition 20. The subsequent characteristicity analysis is separate from this recalled pointwise equivalence.

**Theorem** **12** (Solomonoff kernel: convergence, PSD, and universal-pair equivalence)**.** *The series defining $k_{\mathrm{Sol}}\left(x,y\right)$ in (48) converges absolutely and $0<k_{\mathrm{Sol}}\left(x,y\right)\leq Z\leq 1$. It is positive semidefinite, with either of the equivalent feature representations*

(49) $$\begin{array}{cccc} \phi_{x}\left(\omega \right) & =2^{-K(x|\omega )}\; & \; & \mathrm{in}\;L^{2}\left(\Omega ,\pi \right), \end{array}$$

(50) $$\begin{array}{cccc} \Phi_{x}\left(\omega \right) & =2^{-|\omega |/2}2^{-K(x|\omega )}\; & \; & \mathrm{in}\;\ell^{2}\left(\Omega \right)\;\mathrm{with}\;\mathrm{counting}\;\mathrm{measure}. \end{array}$$

*Moreover, there are machine-dependent constants $0<c_{U}\leq C_{U}<\infty$ such that, for every pair of strings,*

(51) $$c_{U}2^{-K(\langle x,y\rangle )}\leq k_{\mathrm{Sol}}\left(x,y\right)\leq C_{U}2^{-K(\langle x,y\rangle )}.$$

*Equivalently, $-\log_{2}k_{\mathrm{Sol}}\left(x,y\right)=K\left(\langle x,y\rangle \right)+O_{U}\left(1\right)$.*

**Proof.**  Since K(x|ω)≥0 and K(y|ω)≥0 for all x,y,ω, each summand satisfies

(52) $$2^{-|\omega |}\cdot 2^{-K(x|\omega )}\cdot 2^{-K(y|\omega )}\leq 2^{-|\omega |}.$$

Hence,

(53) $$k_{\mathrm{Sol}}\left(x,y\right)\leq \sum_{\omega \in \Omega }2^{-|\omega |}.$$

Because Ω=dom(U) is prefix-free, Kraft’s inequality gives

(54) $$\sum_{\omega \in \Omega }2^{-|\omega |}\leq 1.$$

Therefore, the series converges absolutely and $k_{\mathrm{Sol}}\left(x,y\right)\leq 1$.

To prove strict positivity, it is enough to exhibit one program $\omega^{*}\in \Omega$ contributing a positive summand. Since *U* is universal and Ω is nonempty, choose any fixed halting program $\omega^{*}\in \Omega$. Then,

(55) $$2^{-|\omega^{*}|}\cdot 2^{-K(x|\omega^{*})}\cdot 2^{-K(y|\omega^{*})}>0,$$

because all three exponents are finite. Hence, the corresponding summand is positive.

For the PSD claim, Equations (49) and (50) both give

(56) $$k_{\mathrm{Sol}}\left(x,y\right)={\langle \phi_{x},\phi_{y}\rangle }_{L^{2}\left(\pi \right)}={\langle \Phi_{x},\Phi_{y}\rangle }_{\ell^{2}}.$$

It remains to prove (51). Uniform prefix-freeness of the conditional machine gives, for every ω, $\sum_{x}2^{-K(x|\omega )}\leq 1$. Tonelli’s theorem therefore yields

(57) $$\begin{array}{cc} \sum_{x,y}k_{\mathrm{Sol}}\left(x,y\right)\; & =\sum_{\omega \in \Omega }2^{-|\omega |}\left(\sum_{x}2^{-K(x|\omega )}\right)\left(\sum_{y}2^{-K(y|\omega )}\right), \end{array}$$

(58) $$\begin{array}{cc} \; & \leq Z\leq 1. \end{array}$$

Because K(·∣·) is upper semicomputable uniformly in its arguments and Ω is computably enumerable, $k_{\mathrm{Sol}}$ is a uniformly lower-semicomputable semimeasure on paired strings. Universality of the a priori semimeasure and the coding theorem imply the upper bound $k_{\mathrm{Sol}}\left(x,y\right)\leq C_{U}2^{-K(\langle x,y\rangle )}$.

For the reverse bound, let $p_{x,y}^{*}$ be the shortest prefix program for 〈x,y〉. Then, $p_{x,y}^{*}\in \Omega$, and fixed projection programs can run $U(p_{x,y}^{*})$ and return either component. Hence, $K\left(x|p_{x,y}^{*}\right),K\left(y|p_{x,y}^{*}\right)\leq c$ for a machine-dependent constant *c*. The single term $\omega =p_{x,y}^{*}$ gives

(59) $$k_{\mathrm{Sol}}\left(x,y\right)\geq 2^{-|p_{x,y}^{*}|-2c}=2^{-2c}2^{-K(\langle x,y\rangle )},$$

which proves the lower bound.    □

**Corollary** **3** (Universal-machine robustness)**.** *For optimal universal prefix machines U and V, there are constants $0<c_{UV}\leq C_{UV}<\infty$ such that*

(60) $$c_{UV}k_{\mathrm{Sol},V}\left(x,y\right)\leq k_{\mathrm{Sol},U}\left(x,y\right)\leq C_{UV}k_{\mathrm{Sol},V}\left(x,y\right)$$

*for all finite strings x,y. The equivalence is pointwise and does not transport characteristicity; see Remark 10.*

**Proof.**  Combine Theorem 12 with invariance of prefix complexity.    □

**Corollary** **4** (Normalized Solomonoff kernel)**.** *The kernel*

(61) $${\bar{k}}_{\mathrm{Sol}}\left(x,y\right):=\frac{k_{\mathrm{Sol}}\left(x,y\right)}{\sqrt{k_{\mathrm{Sol}}\left(x,x\right)k_{\mathrm{Sol}}\left(y,y\right)}}$$

*is positive semidefinite and satisfies ${\bar{k}}_{\mathrm{Sol}}\left(x,x\right)=1$. It removes the dominant marginal complexity scale. The ideal normalized kernel remains uncomputable; only separately defined compressor-relative analogues are available for numerical work.*

**Proof.**  Normalize the feature vector $\Phi_{x}$ by its nonzero norm.    □

**Proposition** **4** (Exact Solomonoff characteristicity statement)**.** *The Solomonoff kernel is characteristic on a probability class P if and only if, for every nonzero ν=P−Q with P,Q∈P,*

(62) $$\sum_{\omega \in \Omega }2^{-|\omega |}{\left(\sum_{x}\nu \left(x\right)2^{-K(x|\omega )}\right)}^{2}>0.$$

*The pair-complexity equivalence does not by itself imply this condition, and whether (62) holds is a property of the machine rather than of the construction: see Theorem 13.*

**Proof.**  Apply Proposition 1 to either of the two feature representations in Theorem 12.    □

### 4.5. Characteristicity Is Machine-Dependent

Criterion (62) is stated for a fixed universal prefix machine, and the dependence is not removable. (The two machines of this subsection, and the proofs that they place criterion (62) on opposite sides, are due to an anonymous referee of this paper.) We exhibit two universal conditional prefix machines whose Solomonoff kernels sit on opposite sides of it. Throughout, ϵ is the empty string, *V* is an arbitrary universal conditional prefix machine, and machine-indexed complexities and program sets are written as $K_{W}$ and $\Omega_{W}=\mathrm{dom}\left(W\left(\cdot ,\epsilon \right)\right)$, as in Definition 20.

**Proposition** **5** (A universal machine whose Solomonoff kernel is not characteristic)**.** *Fix distinct strings a≠b and define*

(63) $$U^{-}\left(p,z\right):=\left\{\begin{array}{cc} a, & p=00,\\ b, & p=01,\\ V(q,z), & p=111q,\\ \mathrm{undefined}, & \mathrm{otherwise}. \end{array}\right.$$

*Then, $U^{-}$ is a universal conditional prefix machine and, for $\nu =\delta_{a}-\delta_{b}$,*

(64) $$\sum_{\omega \in \Omega_{U^{-}}}2^{-|\omega |}{\left(\sum_{x}\nu \left(x\right)2^{-K_{U^{-}}\left(x|\omega \right)}\right)}^{2}=0.$$

*Hence, ${\mathrm{MMD}}_{k_{\mathrm{Sol},U^{-}}}\left(\delta_{a},\delta_{b}\right)=0$ although $\delta_{a}\neq \delta_{b}$, and $k_{\mathrm{Sol},U^{-}}$ is not characteristic on any class containing these two laws.*

**Proof.**  *Prefix machine.* For every *z*, the program set is {00,01}∪{111q:q∈domV(·,z)}. The blocks 00, 01, and 111· have pairwise incomparable prefixes and domV(·,z) is prefix-free, so the set is prefix-free; its Kraft mass is at most $\frac{1}{4}+\frac{1}{4}+\frac{1}{8}\leq 1$, as Definition 20 requires.

*Universality.* The branch p=111q gives $K_{U^{-}}\left(x|z\right)\leq K_{V}\left(x|z\right)+3$ for all x,z, so $U^{-}$ is optimal and Theorem 12 and Corollary 3 apply to it.

*The two complexities coincide.* No program of length 0 or 1 halts, and the programs 00 and 01 output *a* and *b* for every auxiliary input. Hence,

(65) $$K_{U^{-}}\left(a|z\right)=K_{U^{-}}\left(b|z\right)=2\;\mathrm{for}\;\mathrm{every}\;z;$$

in particular, for every $\omega \in \Omega_{U^{-}}$.

*Cancellation.* With $\nu =\delta_{a}-\delta_{b}$, the inner sum in (64) equals $2^{-2}-2^{-2}=0$ at every ω, so the energy vanishes term by term. The conclusion follows from Proposition 4.    □

**Proposition** **6** (A universal machine whose Solomonoff kernel is strictly positive definite)**.** *Let $e\left(y\right):=1^{\left|y\right|}0y$ be the standard self-delimiting code, put c(y):=00e(y) and $C:=\{c\left(y\right):y\in {\left\{0,1\right\}}^{*}\}$, and define*

(66) $$U^{+}\left(p,z\right):=\left\{\begin{array}{cc} y, & p=01,\;z=c(y),\\ \epsilon , & p=01,\;z\notin C,\\ y, & p=c(t),\;z=c(y),\\ t, & p=c(t),\;z\notin C,\\ V(q,z), & p=111q,\\ \mathrm{undefined}, & \mathrm{otherwise}. \end{array}\right.$$

*Then, $U^{+}$ is a universal conditional prefix machine and $k_{\mathrm{Sol},U^{+}}$ is strictly positive definite on every finite set: for all distinct $x_{1},\ldots ,x_{n}$ and all real $a_{1},\ldots ,a_{n}$ not all zero, writing $M:=\max_{i}\left|a_{i}\right|>0$ and letting j be an index attaining the maximum,*

(67) $$\sum_{\omega \in \Omega_{U^{+}}}2^{-|\omega |}{\left(\sum_{i=1}^{n}a_{i}2^{-K_{U^{+}}\left(x_{i}|\omega \right)}\right)}^{2}\;\geq \;2^{-|c(x_{j})|}\;\frac{M^{2}}{64}\;>\;0.$$

*The energy is also strictly positive for every nonzero signed measure of finite total variation on the countable string space. In particular, $k_{\mathrm{Sol},U^{+}}$ is characteristic on all probability laws on that space, a statement stronger than the finite-support zero-mass condition.*

**Proof.**  *Prefix machine.* The program set is {01}∪C∪{111q:q∈domV(·,z)}. Its three blocks begin with 01,  00, and 111, and are pairwise incomparable; *C* is prefix-free because *e* is self-delimiting, and domV(·,z) is prefix-free. Since |c(y)|=2|y|+3,

(68) $$\sum_{y\in {\left\{0,1\right\}}^{*}}2^{-|c(y)|}=\frac{1}{8}\sum_{n\geq 0}2^{n}2^{-2n}=\frac{1}{4},$$

so the Kraft mass is at most $\frac{1}{4}+\frac{1}{4}+\frac{1}{8}\leq 1$.

*Universality.* The branch p=111q gives $K_{U^{+}}\left(x|z\right)\leq K_{V}\left(x|z\right)+3$.

*The codes are admissible programs.* Every element of *C* has a length of at least three, so ϵ∉C, and $U^{+}\left(c\left(t\right),\epsilon \right)=t$ halts. Hence, $C\subseteq \Omega_{U^{+}}$ and each c(y) is available as a summation index in (48).

*Complexities at ω=c(y).* The program 01 outputs *y* and no shorter program halts, so $K_{U^{+}}\left(y|c\left(y\right)\right)=2$. For x≠y, neither 01 nor any c(t) outputs *x* at auxiliary input c(y)∈C, so only the simulation branch is available and $K_{U^{+}}\left(x|c\left(y\right)\right)=3+K_{V}\left(x|c\left(y\right)\right)$.

*Lower bound.* Let *j* attain $M=\max_{i}\left|a_{i}\right|>0$ and take $\omega :=c\left(x_{j}\right)\in \Omega_{U^{+}}$. By the previous step,

(69) $$A:=\sum_{i=1}^{n}a_{i}2^{-K_{U^{+}}\left(x_{i}|\omega \right)}=\frac{a_{j}}{4}+\frac{1}{8}\sum_{i\neq j}a_{i}2^{-K_{V}\left(x_{i}|\omega \right)}.$$

Kraft’s inequality for V(·,ω) gives $\sum_{i\neq j}2^{-K_{V}\left(x_{i}|\omega \right)}\leq 1$, so

(70) $$|A|\;\geq \;\frac{|a_{j}|}{4}-\frac{1}{8}\sum_{i\neq j}\left|a_{i}\right|2^{-K_{V}\left(x_{i}|\omega \right)}\;\geq \;\frac{M}{4}-\frac{M}{8}=\frac{M}{8}.$$

Every term of the energy sum is non-negative, so retaining only $\omega =c(x_{j})$ gives $2^{-|c(x_{j})|}A^{2}\geq 2^{-|c(x_{j})|}M^{2}/64$.

*Countable extension.* Let $0\neq \nu \in \ell^{1}\left({\left\{0,1\right\}}^{*}\right)$ and put $M={∥\nu ∥}_{\infty }>0$. This maximum is attained: there are only finitely many strings with |ν(x)|≥M/2, and their maximum must be *M*. Choose such an $x_{j}$. All inner series converge absolutely because the features are bounded by one; moreover, the absolute sum used to exchange the kernel and signed-measure sums is at most ${Z∥\nu ∥}_{1}^{2}<\infty$. At $\omega =c(x_{j})$, the same calculation gives

$$\left|\sum_{x}\nu \left(x\right)2^{-K_{U^{+}}\left(x|\omega \right)}\right|\geq \frac{M}{4}-\frac{1}{8}\sum_{x\neq x_{j}}\left|\nu \left(x\right)\right|2^{-K_{V}\left(x|\omega \right)}\geq \frac{M}{8}.$$

The energy is therefore at least $2^{-|c(x_{j})|}M^{2}/64>0$. Applying this to ν=P−Q proves the asserted countable characteristicity.    □

**Theorem** **13** (Machine dependence of Solomonoff characteristicity)**.** *Characteristicity of the Solomonoff kernel is not an invariant of the construction. By Propositions 5 and 6, there are universal conditional prefix machines $U^{-}$ and $U^{+}$ such that $k_{\mathrm{Sol},U^{-}}$ fails to be characteristic on a two-point set while $k_{\mathrm{Sol},U^{+}}$ has positive energy on every nonzero finite-total-variation signed measure and is characteristic on all countable probability laws. Criterion (62) therefore has no machine-free answer, and any assertion that the Solomonoff kernel is characteristic must name the machine.*

**Remark** **10** (Why this does not conflict with Corollary 3)**.** *Both machines are optimal, so Theorem 12 and Corollary 3 apply to both: the two kernels agree pointwise up to machine-dependent constants. A two-sided pointwise bound $c\;k_{V}\leq k_{U}\leq C\;k_{V}$ gives the corresponding comparison of $\sum_{i,j}\nu_{i}\nu_{j}k\left(x_{i},x_{j}\right)$ when the coefficients have one sign. For mixed-sign coefficients, the form is not monotone in the kernel entries, so that comparison does not follow in general. Characteristicity is a statement about signed zero-mass coefficient vectors and is therefore not transported by the pair-complexity equivalence (51). The two machines make the gap concrete: their kernels agree up to constant factors at every pair of arguments, yet one has a nontrivial null vector on {a,b} and the other has none on any finite set.*

**Remark** **11** (Scope of the machine dependence)**.** *Results that use only positive semidefiniteness or the pair-complexity equivalence are unaffected, since both constructions are legitimate Solomonoff kernels. What changes is the status of Layer-A detection statements: any Layer-A claim that requires injectivity of the mean embedding must now name a machine for which (62) is known to hold, such as $U^{+}$, or be stated conditionally. Nothing at Layer B or Layer C changes. The implementable kernels are the D2KE compressor kernels of Definition 18, whose characteristicity is described by the exact criterion of Theorem 10 and can be certified on finite supports by Theorem 11; neither involves a universal machine. These are identification criteria, not an automatic guarantee for every choice of compressor and landmarks.*

*For diagonal normalization, the finite-support conclusion is immediate: $\bar{G}=D^{-1}GD^{-1}$ with positive invertible $D=\mathrm{diag}(\sqrt{k(x_{i},x_{i})})$ preserves strict positive definiteness. Thus, ${\bar{k}}_{\mathrm{Sol},U^{+}}$ is characteristic on every finite support. The equal feature vectors for a,b under $U^{-}$ have equal norms, so their normalized vectors remain equal. Machine dependence therefore also holds for the normalized kernel on finite supports. This argument does not extend automatically to arbitrary countable laws: division by the feature norm need not preserve the $\ell^{1}$ class of coefficients.*

The following theorem controls program-set truncation of the ideal kernel. It does not make the truncation computable, because its conditional-complexity features and residual halting mass remain uncomputable.

**Theorem** **14** (Uniform Truncation Bound for Solomonoff Kernel)**.** *Let F⊂Ω be finite and define the truncated kernel*

(71) $$k_{F}\left(x,y\right):=\sum_{\omega \in F}2^{-|\omega |}\cdot 2^{-K(x|\omega )}\cdot 2^{-K(y|\omega )}.$$

*Let $\tau \left(F\right):=\sum_{\omega \in \Omega \setminus F}2^{-|\omega |}$ denote the residual Kraft mass. Then, for all strings x,y:*

(72) $$0\leq k_{\mathrm{Sol}}\left(x,y\right)-k_{F}\left(x,y\right)\leq \tau \left(F\right).$$

*Consequently, $\sup_{x,y}\left|k_{\mathrm{Sol}}\left(x,y\right)-k_{F}\left(x,y\right)\right|\leq \tau \left(F\right)$. If $F_{1}\subset F_{2}\subset \ldots$ with ${⋃}_{L=1}^{\infty }F_{L}=\Omega$, then $\sup_{x,y}\left|k_{\mathrm{Sol}}\left(x,y\right)-k_{F_{L}}\left(x,y\right)\right|\to 0$.*

**Proof.**  The difference is

(73) $$k_{\mathrm{Sol}}\left(x,y\right)-k_{F}\left(x,y\right)=\sum_{\omega \in \Omega \setminus F}2^{-|\omega |}\cdot 2^{-K(x|\omega )}\cdot 2^{-K(y|\omega )}.$$

Every summand is non-negative, so the difference is non-negative. Since $2^{-K(x|\omega )}\leq 1$ and $2^{-K(y|\omega )}\leq 1$:

(74) $$k_{\mathrm{Sol}}\left(x,y\right)-k_{F}\left(x,y\right)\leq \sum_{\omega \in \Omega \setminus F}2^{-|\omega |}=\tau \left(F\right).$$

If $F_{L}↑\Omega$, then $\Omega \setminus F_{L}$ decreases to the empty set. Since $\sum_{\omega \in \Omega }2^{-|\omega |}\leq 1$ is summable, continuity from above gives $\tau (F_{L})\to 0$.    □

**Remark** **12** (Connecting Layers A and B)**.** *Theorem 14 and the perturbation theorems control the MMD and HSIC for ideal finite-program approximants when their tail mass is known. They do not turn those approximants into computable compressor kernels: conditional complexity and halting mass remain uncomputable. The separate compressor-to-joint-code result is Theorem 22, under its explicit approximation hypothesis.*

**Proposition** **7** (Computable conditional-feature proxy)**.** *Fix a finite F⊂Ω and a set S of strings. Suppose computable values $\tilde{K}\left(x|\omega \right)$ satisfy*

(75) $$|\tilde{K}\left(x|\omega \right)-K\left(x|\omega \right)|\leq \Delta \;\left(x\in S,\;\omega \in F\right).$$

*Define*

(76) $${\tilde{k}}_{F}\left(x,y\right):=\sum_{\omega \in F}2^{-|\omega |}2^{-\tilde{K}\left(x|\omega \right)}2^{-\tilde{K}\left(y|\omega \right)},\;W_{F}:=\sum_{\omega \in F}2^{-|\omega |}.$$

*Then, ${\tilde{k}}_{F}$ is a computable finite-rank PSD kernel and, uniformly on S×S,*

(77) $$|k_{\mathrm{Sol}}\left(x,y\right)-{\tilde{k}}_{F}\left(x,y\right)|\leq \tau \left(F\right)+W_{F}\left(2^{2\Delta }-1\right).$$

**Proof.**  The proxy is an explicit finite weighted feature inner product. Each proxy feature differs multiplicatively from its ideal feature by a factor in $\left[2^{-\Delta },2^{\Delta }\right]$; hence, each product changes by at most $2^{2\Delta }-1$ in absolute value after using that the ideal product is at most one. Sum over *F* and add the truncation error from Theorem 14.    □

### 4.6. Variational Complexity Interpretation

The variational bound gives a precise meaning to the informal idea that “the Solomonoff kernel value is large when a short shared explanation exists.” Specifically, $-\log_{2}k_{\mathrm{Sol}}\left(x,y\right)$ is at most the cost of the best shared program: the program length |ω| plus the residual complexities K(x|ω) and K(y|ω). If a short program makes both *x* and *y* simple, the kernel value is large.

**Proposition** **8** (One-Sided Variational Upper Bound over Prefix Programs)**.** *For all $x,y\in {\left\{0,1\right\}}^{*}$:*

(78) $$-\log_{2}k_{\mathrm{Sol}}\left(x,y\right)\leq \underset{\omega \in \Omega }{\inf}\left(|\omega |+K(x|\omega )+K(y|\omega )\right),$$

*where Ω=dom(U) is the prefix-free domain of the fixed universal prefix machine U. In particular, for every ω∈Ω:*

(79) $$-\log_{2}k_{\mathrm{Sol}}\left(x,y\right)\leq \left|\omega \right|+K\left(x|\omega \right)+K\left(y|\omega \right).$$

**Proof.**  By definition:

(80) $$k_{\mathrm{Sol}}\left(x,y\right)=\sum_{\omega \in \Omega }2^{-|\omega |}\cdot 2^{-K(x|\omega )}\cdot 2^{-K(y|\omega )}.$$

Fix any ω∈Ω. Since all summands are non-negative:

(81) $$k_{\mathrm{Sol}}\left(x,y\right)\geq 2^{-|\omega |}\cdot 2^{-K(x|\omega )}\cdot 2^{-K(y|\omega )}.$$

Taking negative base-2 logarithms gives:

(82) $$-\log_{2}k_{\mathrm{Sol}}\left(x,y\right)\leq \left|\omega \right|+K\left(x|\omega \right)+K\left(y|\omega \right).$$

Infimizing over ω∈Ω proves the claim.    □

**Remark** **13** (Interpretation of the Variational Bound)**.** *The quantity*

(83) $$\underset{\omega \in \Omega }{\inf}\left(|\omega |+K(x|\omega )+K(y|\omega )\right)$$

*may be interpreted as the cost of a shared prefix description ω together with the residual conditional complexities of x and y relative to that description. Thus, $-\log_{2}k_{\mathrm{Sol}}\left(x,y\right)$ is small when there exists a short prefix program relative to which both x and y are simple.*

**Remark** **14** (Relation to joint complexity)**.** *Theorem 12 proves the stronger identity*

(84) $$-\log_{2}k_{\mathrm{Sol}}\left(x,y\right)=K\left(\langle x,y\rangle \right)+O_{U}\left(1\right).$$

*The separate variational quantity*

(85) $$\underset{\omega \in \Omega }{\inf}\left(|\omega |+K(x|\omega )+K(y|\omega )\right)$$

*is an interpretable best-shared-explanation upper bound, but no equality with joint complexity is needed for the kernel theorem.*

### 4.7. Solomonoff Feature Mixture

Both D2KE and $k_{\mathrm{Sol}}$ are uncentered feature mixtures of the form

(86) $$k\left(x,y\right)=\int \phi_{\omega }\left(x\right)\phi_{\omega }\left(y\right)\;d\pi \left(\omega \right).$$

This is the only Solomonoff feature-mixture convention used here. Centered variants are different kernels and are not required for the MMD, HSIC, or the discrete KSD developed below; see Part IV [4].

### 4.8. Alternative Positive Semidefinite Realizations of the Same Geometry

The discrepancy interface requires a PSD kernel; it does not require a catalogue of every possible PSD repair. Table 2 records the alternatives needed to delimit the scope of the theory. D2KE and the Solomonoff kernel are the intrinsic constructions used in the main results.

Direct exponentiation, MDS, diffusion, clipping, shifting, and shrinkage are standard kernel constructions. They may be useful computationally, but they do not add an AIT-specific theorem beyond the assumptions in the table. Their MMD and HSIC consequences are the standard RKHS consequences of PSD and characteristicity.

## 5. Direction II: From Kernel Discrepancies to Algorithmic Information

Section 4 turned algorithmic dissimilarities into kernels. This section runs the interface backwards and asks what the standard discrepancies of Section 3 mean once those kernels are in place. The asymmetry flagged in Section 1 appears here in concrete form: the identities we obtain are exact, but the connections to genuinely algorithmic quantities are one-sided, and their converses require support and eigenvalue hypotheses that we state rather than assume. Figure 3 maps the dependencies.

This section develops the reverse direction: interpreting kernel discrepancy measures through the lens of algorithmic information theory.

### 5.1. The HSIC as the Product-Kernel MMD on Countable Spaces

Let *k* and *l* be bounded PSD kernels on countable spaces X and Y. Their product kernel is

(87) $$q\left(\left(x,y\right),\left(x^{\prime },y^{\prime }\right)\right):=k\left(x,x^{\prime }\right)l\left(y,y^{\prime }\right).$$

**Proposition** **9** (The HSIC as a signed product-kernel energy)**.** *With $\eta :=P_{XY}-P_{X}⊗P_{Y}$,*

(88) $${\mathrm{HSIC}}_{k,l}\left(P_{XY}\right)={∥\mu_{q}\left(\eta \right)∥}_{H_{k}⊗H_{l}}^{2}={\mathrm{MMD}}_{q}^{2}\left(P_{XY},P_{X}⊗P_{Y}\right).$$

**Proof.**  The product feature is ϕ(x)⊗ψ(y). Its mean under the joint law minus its mean under the product law is the cross-covariance tensor. Its squared norm is the HSIC.    □

The following is the countable-discrete specialization of Szabó and Sriperumbudur [35] (Theorem 3(i)). We include a direct proof to make the zero-marginal signed-measure argument explicit.

**Theorem** **15** (Characteristic marginals detect independence on countable spaces)**.** *If k and l are characteristic on all probability measures on their respective countable spaces, then*

(89) $${\mathrm{HSIC}}_{k,l}\left(P_{XY}\right)=0\;⟺\;P_{XY}=P_{X}⊗P_{Y}.$$

**Proof.**  Independence makes η=0. Conversely, suppose $\mu_{q}\left(\eta \right)=0$. The signed measure η has zero marginals. For $g\in H_{l}$, define $\nu_{g}\left(x\right):=\sum_{y}\eta \left(x,y\right)g\left(y\right)$. Boundedness of *l* makes *g* bounded, so $\nu_{g}$ has finite variation; its total mass is zero. Taking the partial inner product of $\mu_{q}\left(\eta \right)=0$ with *g* gives $\mu_{k}\left(\nu_{g}\right)=0$. Characteristicity of *k* and Proposition 1 therefore give $\nu_{g}=0$.

Fix *x* and write $r_{x}\left(y\right):=\eta \left(x,y\right)$. This row is a finite zero-mass signed measure. The equality $\nu_{g}\left(x\right)=0$ for every $g\in H_{l}$ says $\mu_{l}\left(r_{x}\right)=0$, so characteristicity of *l* gives $r_{x}=0$. Every row of η is zero, hence η=0.    □

**Remark** **15.** *The theorem proves the property needed by the HSIC. It does not assert that k⊗l is characteristic on every probability measure on the product space; it asserts that it separates the zero-marginal signed measures generated by departures from independence.*

### 5.2. Compressor Proxy for AMI

**Definition** **21** (Compressor-Based Dependence Proxy)**.** *For a compressor C with code-length function $\ell_{C}$:*

(90) $$I_{C}^{\mathrm{pair}}\left(x;y\right):=\ell_{C}\left(x\right)+\ell_{C}\left(y\right)-\ell_{C}^{\mathrm{pair}}\left(x,y\right),$$

*using the self-delimiting, order-symmetrized paired length of Definition 8.*

This is a compressor-relative shared-code score. Its relation to AMI is conditional on the following explicit approximation hypothesis; no such uniform hypothesis is asserted here for gzip, LZMA, or another named compressor.

**Theorem** **16** (Compressor Proxy Approximates AMI)**.** *Fix a class $C_{n}\subset {\left\{0,1\right\}}^{n}$. Assume compressor C satisfies:*

(91) $$\begin{array}{cc} \underset{x\in C_{n}}{\sup}\left|\ell_{C}\left(x\right)-K\left(x\right)\right| & \leq \varepsilon (n), \end{array}$$

(92) $$\begin{array}{cc} \underset{\left(x,y\right)\in C_{n}^{2}}{\sup}\left|\ell_{C}^{\mathrm{pair}}\left(x,y\right)-A_{K}\left(x,y\right)\right|\; & \leq \varepsilon (n), \end{array}$$

*where $A_{K}\left(x,y\right)$ is the symmetrized paired complexity from Theorem 22. Then, for all $\left(x,y\right)\in C_{n}^{2}$:*

(93) $$|I_{C}^{\mathrm{pair}}\left(x;y\right)-\mathrm{AMI}\left(x;y\right)|\leq 3\varepsilon \left(n\right)+O_{U}\left(1\right).$$

**Proof.**  (94) $$\begin{array}{cc} |I_{C}^{\mathrm{pair}}\left(x;y\right)-\left[K\left(x\right)+K\left(y\right)-A_{K}\left(x,y\right)\right]|\; & \leq |\ell_{C}\left(x\right)-K\left(x\right)|+|\ell_{C}\left(y\right)-K\left(y\right)|, \end{array}$$

(95) $$\begin{array}{cc} \; & \;+|\ell_{C}^{\mathrm{pair}}\left(x,y\right)-A_{K}\left(x,y\right)|, \end{array}$$

(96) $$\begin{array}{cc} \; & \leq 3\varepsilon (n). \end{array}$$

The two fixed pair orders have complexities differing by $O_{U}\left(1\right)$, so $A_{K}\left(x,y\right)=K\left(\langle x,y\rangle \right)+O_{U}\left(1\right)$, which completes the proof.    □

**Remark** **16.** *This theorem does not assert that the HSIC equals $E[I_{C}\left(X;Y\right)]$. Rather, it provides a pointwise approximation guarantee: if the compressor well-approximates Kolmogorov complexity on the stated finite family, then the joint-code score approximates AMI. The HSIC remains a separate dependence statistic built from PSD marginal kernels; it is not the expectation of this score.*

### 5.3. Independence and Small AMI

**Theorem** **17** (Expected algorithmic MI and the HSIC bridge)**.** *Let $f=P_{XY}$ be a computable probability mass function on pairs of finite strings, let $I_{\mathrm{Sh}}\left(X;Y\right)$ be Shannon mutual information in bits, and write*

(97) $$I_{U}\left(x:y\right):=K\left(x\right)+K\left(y\right)-K\left(\langle x,y\rangle \right).$$

*Then, the standard expectation theorem for algorithmic mutual information gives*

(98) $$I_{\mathrm{Sh}}\left(X;Y\right)-K\left(f\right)-O_{U}\left(1\right)\leq E_{f}I_{U}\left(X:Y\right)\leq I_{\mathrm{Sh}}\left(X;Y\right)+2K\left(f\right)+O_{U}\left(1\right).$$

*If a finite program computing the source f is supplied as auxiliary information (denoted by f in the conditional complexities), then*

(99) $$E_{f}I_{U}\left(X:Y|f\right)=I_{\mathrm{Sh}}\left(X;Y\right)+O_{U}\left(1\right),$$

*where $I_{U}\left(x:y|f\right):=K\left(x|f\right)+K\left(y|f\right)-K\left(\langle x,y\rangle |f\right)$. If $k\left(x,x\right)\leq \kappa_{X}^{2}$ and $l\left(y,y\right)\leq \kappa_{Y}^{2}$, then*

(100) $${\mathrm{HSIC}}_{k,l}\left(P_{XY}\right)\leq 2\kappa_{X}^{2}\kappa_{Y}^{2}\left(\ln2\right)I_{\mathrm{Sh}}\left(X;Y\right).$$

*Consequently, conditional expected AMI controls the HSIC up to the universal additive constant in (99); the unconditional version additionally pays the source-description terms in (98).*

**Proof.**  The expectation statements are the expected-algorithmic-mutual-information theorem of [37] (Section 3.3). For the HSIC bound, set $R=P_{XY}-P_{X}⊗P_{Y}$ and use the product feature Ψ(x,y)=k(x,·)⊗l(y,·), whose norm is at most $\kappa_{X}\kappa_{Y}$. Since the HSIC is the product-kernel MMD,

(101) $$\begin{array}{cc} \sqrt{{\mathrm{HSIC}}_{k,l}}\; & =∥\int \Psi \;dR∥\leq \kappa_{X}\kappa_{Y}{∥R∥}_{\mathrm{TV}}, \end{array}$$

(102) $$\begin{array}{cc} \; & =2\kappa_{X}\kappa_{Y}\mathrm{TV}\left(P_{XY},P_{X}⊗P_{Y}\right). \end{array}$$

Squaring and applying Pinsker’s inequality gives $\mathrm{HSIC}\leq 2\kappa_{X}^{2}\kappa_{Y}^{2}D_{\mathrm{KL}}\left(P_{XY}∥P_{X}⊗P_{Y}\right)$. Finally, $D_{\mathrm{KL}}=\left(\ln2\right)I_{\mathrm{Sh}}$ for mutual information in bits.    □

**Remark** **17** (Pointwise versus expected independence)**.** *Equations (98) and (99) are expectation statements for a computable source. They do not imply that every pair sampled independently has small pointwise algorithmic mutual information, nor that a numerical HSIC value estimates AMI. Conversely, a small HSIC can control expected AMI only under an independence-characteristic product kernel and quantitative lower bounds that are uniform over the source class.*

**Remark** **18** (Scope)**.** *The bridge is one-sided and source-relative: expected AMI controls Shannon MI up to K(f), Shannon MI controls total variation, and total variation controls the HSIC. This is distinct from the pointwise compressor proxy in Theorem 16.*

**Theorem** **18** (Support- and kernel-dependent two-sided comparison)**.** *Let $X=\{x_{1},\ldots ,x_{r}\}$ and $Y=\{y_{1},\ldots ,y_{s}\}$ after removing zero-marginal states, with r,s≥2, and let k,l be bounded PSD kernels with $\sup_{x}k\left(x,x\right)\leq \kappa_{X}^{2}$ and $\sup_{y}l\left(y,y\right)\leq \kappa_{Y}^{2}$. **Assume** k **is characteristic on X and** l **is characteristic on Y** in the sense of Proposition 2. Let $G_{k}\in R^{r\times r}$ and $G_{l}\in R^{s\times s}$ be the marginal Gram matrices, and define*

(103) $$D:=\left\{A\in R^{r\times s}:A1_{s}=0,A^{⊤}1_{r}=0\right\},\;\lambda_{D}:=\min_{A\in {D,\;∥A∥}_{F}=1}\mathrm{tr}\left(G_{k}AG_{l}A^{⊤}\right),$$

(104) $$r_{\min}:=\min_{x,y}P_{X}\left(x\right)P_{Y}\left(y\right)>0.$$

*Write $V_{r}:=\left\{u\in R^{r}:1_{r}^{⊤}u=0\right\}$, and let $\alpha_{k}:=\lambda_{\min}\left(G_{k}{|}_{V_{r}}\right)$, $\beta_{l}:=\lambda_{\min}\left(G_{l}{|}_{V_{s}}\right)$ be the minimum eigenvalues of the Gram matrices restricted to the zero-sum subspaces. Then,*

(105) $$\lambda_{D}=\alpha_{k}\;\beta_{l},$$

*so $\lambda_{D}>0$ if and only if both marginal kernels are characteristic on their supports, which the hypothesis guarantees. Moreover,*

(106) $$\lambda_{D}r_{\min}\left(\ln2\right)I_{\mathrm{Sh}}\left(X;Y\right)\leq {\mathrm{HSIC}}_{k,l}\left(P_{XY}\right)\leq 2\kappa_{X}^{2}\kappa_{Y}^{2}\left(\ln2\right)I_{\mathrm{Sh}}\left(X;Y\right).$$

*These bounds give a uniform two-sided comparison on finite model classes for which the restricted eigenvalue and product marginal masses are uniformly bounded away from zero. If either marginal support is a singleton, both the HSIC and Shannon mutual information are zero; the spectral constants above are not defined or needed in that degenerate case.*

**Proof.**  We first prove (105). Identify $R^{r\times s}$ with $R^{r}⊗R^{s}$ via $A\mapsto \sum_{ij}A_{ij}e_{i}⊗f_{j}$; the Frobenius inner product becomes the tensor inner product. Under this identification, $\mathrm{tr}\left(G_{k}AG_{l}A^{⊤}\right)={\langle A,\left(G_{k}⊗G_{l}\right)A\rangle }_{F}$, and $D=V_{r}⊗V_{s}$. The restriction of $G_{k}⊗G_{l}$ to $V_{r}⊗V_{s}$ is $\left(G_{k}{|}_{V_{r}}\right)⊗\left(G_{l}{|}_{V_{s}}\right)$, whose spectrum is the set of pairwise products of the two restricted spectra. Both restrictions are PSD, so the minimum of the product spectrum is $\alpha_{k}\beta_{l}$, giving (105). By Proposition 2, characteristicity of *k* on X is exactly $\alpha_{k}>0$, and likewise for *l*; hence, $\lambda_{D}>0$ under the stated hypothesis.

For the bounds, put $R=P_{X}⊗P_{Y}$ and $A\left(x,y\right):=P_{XY}\left(x,y\right)-R\left(x,y\right)$. Then, A∈D and

(107) $$\mathrm{HSIC}=\mathrm{tr}\left(G_{k}AG_{l}A^{⊤}\right)\geq \lambda_{D}{∥A∥}_{F}^{2}.$$

Since logu≤u−1,

(108) $$D_{\mathrm{KL}}\left(P_{XY}∥R\right)\leq \chi^{2}\left(P_{XY},R\right)=\sum_{x,y}\frac{A{\left(x,y\right)}^{2}}{R(x,y)}\leq \frac{{∥A∥}_{F}^{2}}{r_{\min}}.$$

Therefore, ${∥A∥}_{F}^{2}\geq r_{\min}\left(\ln2\right)I_{\mathrm{Sh}}$. The upper bound is Theorem 17.    □

**Remark** **19** (No universal converse)**.** *Multiplying one characteristic marginal kernel by a positive constant preserves characteristicity but rescales the HSIC while leaving mutual information unchanged. Even with fixed diagonal normalization, $\lambda_{D}$ and $r_{\min}$ may approach zero. A kernel-independent converse is therefore impossible without explicit spectral, support, and normalization conditions.*

### 5.4. MMD and Compressibility Profiles

**Definition** **22** (Compressibility Profile)**.** *Fix a non-negative compressor-relative dissimilarity $d_{C}$; for example, $d_{C}\left(x,\omega \right)=\max\left\{0,{\mathrm{NCD}}_{C}\left(x,\omega \right)\right\}$. For a distribution P and bandwidth γ, the* compressibility profile *is the function $\rho_{P}^{\gamma }:\Lambda \to \left[0,1\right]$ defined by:*

(109) $$\rho_{P}^{\gamma }\left(\omega \right):=E_{X∼P}\left[e^{-\gamma d_{C}\left(X,\omega \right)}\right].$$

The compressibility profile $\rho_{P}^{\gamma }\left(\omega \right)$ answers a simple question: how well does a typical sample from *P* compress relative to the landmark ω? The following theorem shows that the MMD with D2KE kernels is exactly the $L^{2}$ distance between these profiles. Two distributions are indistinguishable (MMD=0) if and only if they compress identically relative to π-almost every landmark—a natural algorithmic notion of distributional equality.

**Theorem** **19** (MMD as Compressibility-Profile Distance)**.** *For D2KE kernels with the non-negative dissimilarity $d=d_{C}$ above:*

*(i)*  *${\mathrm{MMD}}_{k_{D2\mathrm{KE}}}\left(P,Q\right)=0$ if and only if $\rho_{P}^{\gamma }\left(\omega \right)=\rho_{Q}^{\gamma }\left(\omega \right)$ for π-almost every ω.*
*(ii)*  *Under the $L^{2}\left(\pi \right)$-determining condition of Definition 19, $\rho_{P}^{\gamma }=\rho_{Q}^{\gamma }$ implies that P=Q.*
*(iii)*  *Without the separating condition, the MMD=0 implies only equality of compressibility profiles, not necessarily that P=Q.*

**Proof.**  **Part (i):** By Theorem 9:

(110) $${\mathrm{MMD}}_{k_{D2\mathrm{KE}}}^{2}\left(P,Q\right)={∥\mu_{P}-\mu_{Q}∥}_{L^{2}\left(\pi \right)}^{2}.$$

The kernel mean embedding is

(111) $$\mu_{P}\left(\omega \right)=E_{X∼P}\left[e^{-\gamma d_{C}\left(X,\omega \right)}\right]=\rho_{P}^{\gamma }\left(\omega \right).$$

Therefore,

(112) $${\mathrm{MMD}}_{k_{D2\mathrm{KE}}}^{2}\left(P,Q\right)=\int_{\Lambda }{\left(\rho_{P}^{\gamma }\left(\omega \right)-\rho_{Q}^{\gamma }\left(\omega \right)\right)}^{2}\;d\pi \left(\omega \right).$$

This equals zero if and only if $\rho_{P}^{\gamma }=\rho_{Q}^{\gamma }$ for π-almost every ω.

**Part (ii):** Follows directly from Theorem 10.

**Part (iii):** Without the determining condition, two different distributions could have identical compressibility profiles (like having identical moments but different distributions).    □

**Remark** **20** (Interpretation)**.** *This theorem shows that the MMD with D2KE kernels measures distributional differences through the lens of compression: two distributions are indistinguishable (have zero MMD) if and only if they compress identically relative to π-almost every landmark in the feature family. This gives an algorithmic interpretation of the kernel two-sample test.*

### 5.5. A Countable-Space Markov–Stein/KSD Interface

The stationary-operator approach is classical: Bresler and Nagaraj [38] (Section 2.2) use I−T as a characterizing operator for a finite irreducible chain; see also Yang et al. [9] for general discrete Stein constructions. We specialize the operator to bounded kernel features on a countable space and make its one-step MMD interpretation explicit.

Let $T_{P}$ be a Markov kernel on a countable space X, and assume that *P* is stationary. For bounded *f*, define

(113) $$\left(T_{P}f\right)\left(x\right):=\sum_{z}T_{P}\left(x,z\right)f\left(z\right),\;A_{P}f:=T_{P}f-f.$$

Stationarity gives $E_{P}A_{P}f=0$.

**Theorem** **20** (Unrestricted Markov–Stein discrepancy)**.** *For every probability measure Q,*

(114) $$\underset{{∥f∥}_{\infty }\leq 1}{\sup}\left|E_{Q}\left[A_{P}f\right]\right|=2\;\mathrm{TV}\left(QT_{P},Q\right).$$

**Proof.**  Writing $\nu =QT_{P}-Q$ gives $E_{Q}A_{P}f=\sum_{z}f\left(z\right)\nu \left(z\right)$. On a countable space, the supremum is attained by f(z)=signν(z) and equals $\sum_{z}\left|\nu \left(z\right)\right|=2\;\mathrm{TV}\left(QT_{P},Q\right)$ under the convention of Remark 4.    □

Let *k* be bounded and PSD, with $k\left(x,x\right)\leq \kappa_{k}^{2}$. Define the Stein feature

(115) $$\xi_{P}\left(x\right):=\sum_{z}T_{P}\left(x,z\right)k\left(z,\cdot \right)-k\left(x,\cdot \right)$$

and

(116) $${\mathrm{KSD}}_{T_{P},k}\left(Q\right):={∥E_{X∼Q}\xi_{P}\left(X\right)∥}_{H_{k}}.$$

**Remark** **21** (Notation: the discrepancy depends on $T_{P}$, not on *P* alone)**.** *A target law does not determine a Markov kernel. Every T with PT=P is admissible, and changing T can change the Stein feature (115) and the resulting discrepancy, so we write ${\mathrm{KSD}}_{T_{P},k}$ rather than ${\mathrm{KSD}}_{P,k}$, with $T_{P}$ denoting the particular target-stationary kernel that has been selected. The dependence is not cosmetic: the detection statement (120) requires P to be the* unique *stationary law of the selected $T_{P}$, which is a property of $T_{P}$ and not of P. The identity operator gives zero discrepancy for every law. However, shrinking the discrepancy by a positive scalar need not change power after null calibration; see Remark 29.*

**Theorem** **21** (Exact Markov KSD–MMD identity)**.** *For every probability measure Q,*

(117) $${\mathrm{KSD}}_{T_{P},k}\left(Q\right)={\mathrm{MMD}}_{k}\left(QT_{P},Q\right).$$

*Moreover, with *

(118) $$\begin{array}{cc} h_{P}\left(x,y\right)\; & :={\langle \xi_{P}\left(x\right),\xi_{P}\left(y\right)\rangle }_{H_{k}}=\left(A_{P}^{x}A_{P}^{y}k\right)\left(x,y\right), \end{array}$$

*$h_{P}$ is PSD, $|h_{P}\left(x,y\right)|\leq 4\kappa_{k}^{2}$, and *

(119) $${\mathrm{KSD}}_{T_{P},k}^{2}\left(Q\right)=E_{X,Y\overset{\mathrm{iid}}{∼}Q}h_{P}\left(X,Y\right).$$

*If k is characteristic on the relevant probabilities and P is the unique stationary law of $T_{P}$, then*

(120) $${\mathrm{KSD}}_{T_{P},k}\left(Q\right)=0\;⟺\;Q=P.$$

*Aperiodicity is not needed for this population statement.*

**Proof.**  Taking the expectation of the Stein feature gives

(121) $$E_{Q}\xi_{P}\left(X\right)=\mu_{k}\left(QT_{P}\right)-\mu_{k}\left(Q\right),$$

which proves (117). The definition of $h_{P}$ makes it the Gram kernel of $\xi_{P}$, proving PSD and (119). Since $∥\xi_{P}\left(x\right)∥\leq 2\kappa_{k}$, Cauchy–Schwarz gives the displayed bound. Finally, zero KSD and characteristicity imply that $QT_{P}=Q$; uniqueness of the stationary law then gives Q=P. The converse follows from stationarity.    □

**Proposition** **10** (D2KE Stein-profile identity)**.** *For D2KE features $f_{\omega }\left(x\right)=e^{-\gamma d(x,\omega )}$,*

(122) $${\mathrm{KSD}}_{T_{P},k_{D2\mathrm{KE}}}^{2}\left(Q\right)=\int_{\Lambda }{\left[E_{Q}\left(T_{P}f_{\omega }-f_{\omega }\right)\right]}^{2}d\pi \left(\omega \right).$$

*Thus, “algorithmic” describes the selected feature geometry; the Stein identity itself remains target-relative through $T_{P}$.*

**Proof.**  Apply the D2KE MMD profile identity to $QT_{P}$ and *Q*, then use Theorem 21.    □

**Proposition** **11** (KSD approximation stability)**.** *If $\sup_{x,y}\left|k\left(x,y\right)-\tilde{k}\left(x,y\right)\right|\leq \varepsilon_{k}$, then*

(123) $$|{\mathrm{KSD}}_{T_{P},k}^{2}\left(Q\right)-{\mathrm{KSD}}_{T_{P},\tilde{k}}^{2}\left(Q\right)|\leq 4\varepsilon_{k}.$$

*If instead $k\left(x,x\right)\leq \kappa_{k}^{2}$ and $\sup_{x}\mathrm{TV}\left(T_{P}\left(x,\cdot \right),{\tilde{T}}_{P}\left(x,\cdot \right)\right)\leq \varepsilon_{T}$, then*

(124) $$|{\mathrm{MMD}}_{k}^{2}\left(QT_{P},Q\right)-{\mathrm{MMD}}_{k}^{2}\left(Q{\tilde{T}}_{P},Q\right)|\leq 8\kappa_{k}^{2}\varepsilon_{T}.$$

**Proof.**  The first claim applies the 4ε MMD-squared perturbation bound to the pair $\left(QT_{P},Q\right)$. For the second, $\mathrm{TV}\left(QT_{P},Q{\tilde{T}}_{P}\right)\leq \varepsilon_{T}$; hence, their mean embeddings differ by at most $2\kappa_{k}\varepsilon_{T}$. Apply ${|∥a∥}^{2}-{∥b∥}^{2}|\leq (∥a∥+∥b∥)∥a-b∥$ and $∥a∥,∥b∥\leq 2\kappa_{k}$.    □

For $X_{1},\ldots ,X_{n}\overset{\mathrm{iid}}{∼}Q$, the biased and unbiased estimators are

(125) $$\begin{array}{cc} {\hat{\mathrm{KSD}}}_{b}^{2}\; & :=\frac{1}{n^{2}}\sum_{i,j=1}^{n}h_{P}\left(X_{i},X_{j}\right)={∥\frac{1}{n}\sum_{i=1}^{n}\xi_{P}\left(X_{i}\right)∥}_{H_{k}}^{2}, \end{array}$$

(126) $$\begin{array}{cc} {\hat{\mathrm{KSD}}}_{u}^{2}\; & :=\frac{1}{n(n-1)}\sum_{i\neq j}h_{P}\left(X_{i},X_{j}\right). \end{array}$$

The first is non-negative; the second is unbiased for ${\mathrm{KSD}}_{T_{P},k}^{2}\left(Q\right)$ and may be negative in a finite sample.

**Proposition** **12** (KSD U-statistic concentration)**.** *If $|h_{P}\left(x,y\right)|\leq H$, then*

(127) $$\mathrm{Pr}\;\left(|{\hat{\mathrm{KSD}}}_{u}^{2}-{\mathrm{KSD}}_{T_{P},k}^{2}\left(Q\right)|>t\right)\leq 2\exp\;\left(-\frac{nt^{2}}{8H^{2}}\right).$$

*In particular, $H=4\kappa_{k}^{2}$ is valid by Theorem 21.*

**Proof.**  Changing one observation affects 2(n−1) ordered summands, each by at most 2H, so the statistic changes by at most 4H/n. McDiarmid’s inequality gives (127).    □

## 6. The Computability Gap: Propagating Error Across Both Directions

Both directions of the interface have now been developed at the ideal layer. Every construction in Section 4 and every identity in Section 5 is exact, and every one of them refers to objects that cannot be computed. This section makes the passage to computable surrogates explicit and tracks what it costs. The results are of one shape: a hypothesis bounding the code-length error, and a conclusion bounding the induced error in the discrepancy. None of them asserts that any named compressor satisfies the hypothesis.

The ideal objects appearing in this paper—Kolmogorov complexity, Solomonoff semimeasures, and kernels defined directly from them—are not computable. This section discusses the gap between theory and practice and provides guidance for practical implementations.

### 6.1. Two-Layer Reading

The main theorems should be read at two levels:

**Level 1 (Ideal theory):** The theorems identify mathematically canonical objects and prove structural facts about them: positive semidefiniteness, complexity bounds, characteristicity under stated assumptions, and exact discrepancy identities. At this level, the statements belong to the theory of ideal algorithmic information.

**Level 2 (Practical motivation):** The theorems motivate practical surrogates obtained by replacing ideal code lengths with compressor lengths produced by concrete schemes (gzip, LZMA, PPM, neural compressors). At this practical level, the present paper does not prove uniform approximation guarantees from compressor-based kernels to their ideal AIT counterparts. Instead, it provides a principled design template and a set of hypotheses that future approximation theory should target.

### 6.2. Practical Implementation Template

A practical implementation proceeds as follows.

The template has four steps. One chooses a compressor *C* and encodes each object as a finite binary string. One builds a dissimilarity $d_{C}$ from code lengths, either the normalized compression distance $d_{C}\left(x,y\right)=\left(\ell_{C}^{\mathrm{pair}}\left(x,y\right)-\min\left\{\ell_{C}\left(x\right),\ell_{C}\left(y\right)\right\}\right)/\max\left\{\ell_{C}\left(x\right),\ell_{C}\left(y\right)\right\}$ or the conditional proxy $d_{C}\left(x|\omega \right)=\ell_{C}\left({\langle \omega ,x\rangle }_{C}\right)-\ell_{C}\left(\omega \right)$. Negative dissimilarity values, if any, are replaced by zero to satisfy Definition 18. One converts $d_{C}$ into a kernel by the landmark construction of Definition 18, using a finite landmark set S⊂Λ,

(128) $$k_{C}\left(x,y\right)=\frac{1}{\left|S\right|}\sum_{\omega \in S}e^{-\gamma d_{C}\left(x,\omega \right)}e^{-\gamma d_{C}\left(y,\omega \right)},$$

which is positive semidefinite by Theorem 9 whatever $d_{C}$ does. Finally, one computes the empirical discrepancy of interest from $k_{C}$. Every step is computable. For the clipped joint-code version, Theorem 22 bounds its difference from a D2KE kernel built from ideal joint-code dissimilarities with the same landmark measure, under the explicit code-length approximation hypothesis. It supplies no general bound to the Solomonoff kernel or for the conditional proxy.

**Remark** **22** (Interpretation caveat)**.** *This distinction is essential. The present theory justifies the architectural route from compression to kernels, but it does not establish that every practical compressor preserves the inferential properties of the ideal kernel. Empirical success of gzip-, LZMA-, or neural-compressor-based kernels should be viewed as evidence for the usefulness of the selected compressor features, not as a theorem about universality.*

### 6.3. Error Propagation

The next result deliberately separates the joint-code proxy from NID. This avoids silently replacing symmetry of information, which carries a logarithmic remainder, by an exact identity.

**Theorem** **22** (Joint-code proxy error and D2KE propagation)**.** *Fix the paired serialization from Definition 8, and put*

(129) $$\begin{array}{cccc} A_{K}\left(x,y\right) & :=\min\{K\left({\langle x,y\rangle }_{C}\right),K\left({\langle y,x\rangle }_{C}\right)\},\; & A_{C}\left(x,y\right) & :=\ell_{C}^{\mathrm{pair}}\left(x,y\right), \end{array}$$

(130) $$\begin{array}{cccc} J_{K}\left(x,y\right) & :=\frac{A_{K}\left(x,y\right)-\min\left\{K\left(x\right),K\left(y\right)\right\}}{\max\{K(x),K(y)\}},\; & J_{C}\left(x,y\right) & :=\frac{A_{C}\left(x,y\right)-\min\left\{\ell_{C}\left(x\right),\ell_{C}\left(y\right)\right\}}{\max\{\ell_{C}\left(x\right),\ell_{C}\left(y\right)\}}. \end{array}$$

*Suppose every marginal and paired code length above is within* Δ *of its corresponding prefix complexity, let L=min{K(x),K(y)}>0, and assume Δ≤L/2. For a constant $c_{U}$ depending only on the fixed machine and serializer,*

(131) $$|J_{C}\left(x,y\right)-J_{K}\left(x,y\right)|\leq \frac{6\Delta }{L}+\frac{2c_{U}\Delta }{L^{2}}.$$

*Furthermore,*

(132) $$J_{K}\left(x,y\right)=\mathrm{NID}\left(x,y\right)+O\;\left(\frac{\log(1+\max\{K(x),K(y)\})}{\max\{K(x),K(y)\}}\right).$$

*Let $d_{C},d_{K}$ be the versions of $J_{C},J_{K}$ clipped below at zero, so that $d_{C},d_{K}\in \left[0,\infty \right)$, and suppose that*

(133) $$\underset{x,\omega }{\sup}\left|d_{C}\left(x,\omega \right)-d_{K}\left(x,\omega \right)\right|\leq \varepsilon .$$

*For D2KE kernels built with the same landmark measure π on* Λ *and features $e^{-\gamma d}$,*

(134) $$\underset{x,y}{\sup}\left|k_{C}^{D2\mathrm{KE}}\left(x,y\right)-k_{K}^{D2\mathrm{KE}}\left(x,y\right)\right|\leq 2\gamma \varepsilon \;\pi \left(\Lambda \right).$$

*Consequently, for any P,Q,*

(135) $$|{\mathrm{MMD}}_{k_{C}}^{2}\left(P,Q\right)-{\mathrm{MMD}}_{k_{K}}^{2}\left(P,Q\right)|\leq 8\gamma \varepsilon \;\pi \left(\Lambda \right).$$

*The factor π(Λ) is not removable: Definition 18 allows any finite positive landmark measure, and for π(Λ)=100, γ=1, ε=0.01, one finds numerically that $\sup_{x,y}\left|k_{C}-k_{K}\right|\approx 0.77>2\gamma \varepsilon =0.02$. It disappears exactly when π is a probability measure.*

**Proof.**  Write $N=A_{K}-\min\left\{K\left(x\right),K\left(y\right)\right\}$ and $N^{\prime }=A_{C}-\min\left\{\ell_{C}\left(x\right),\ell_{C}\left(y\right)\right\}$, and let $D,D^{\prime }$ be the corresponding maxima. Then, $|N^{\prime }-N|\leq 2\Delta$, $|D^{\prime }-D|\leq \Delta$, and $D^{\prime }\geq L/2$. Self-delimiting composition gives $A_{K}\leq K\left(x\right)+K\left(y\right)+c_{U}$, while fixed projections give $A_{K}\geq \max\left\{K\left(x\right),K\left(y\right)\right\}-c_{U}$ after enlarging the same constant. Hence, $\left|N\right|\leq D+c_{U}$. Therefore,

(136) $$\begin{array}{cc} \left|\frac{N^{\prime }}{D^{\prime }}-\frac{N}{D}\right|\; & \leq \frac{2\Delta }{D^{\prime }}+\frac{(D+c_{U})\Delta }{DD^{\prime }}\leq \frac{6\Delta }{L}+\frac{2c_{U}\Delta }{L^{2}}. \end{array}$$

Equation (132) is symmetry of information for prefix complexity. Clipping is nonexpansive, and $|e^{-\gamma a}-e^{-\gamma b}\left|\leq \gamma |a-b\right|$ on [0,∞). Thus, each D2KE feature changes by at most γε; the product changes by at most 2γε, proving (134). The four expectations in MMD^2^ then give the final bound.    □

**Remark** **23.** *The hypothesis is a finite-family approximation assumption, not a claim that an off-the-shelf compressor uniformly approximates Kolmogorov complexity. Together with Theorem 24, the same pointwise D2KE bound also controls the induced HSIC error.*

### 6.4. Perturbation Theorems for the MMD and HSIC

The preceding results bound pointwise kernel approximation error. The following theorems show how such errors propagate into the discrepancy measures of the MMD and HSIC, providing the missing link between Layers A and C.

Intuition is straightforward: MMD^2^ is an expectation of kernel values (a sum of three terms), so if every kernel evaluation changes by at most δ, the overall MMD^2^ changes by at most 4δ. For the HSIC, the argument is similar but involves a product of two centered kernels, so the error bound picks up the kernel bounds $B_{k},B_{l}$ as prefactors. These theorems are the formal reason we can use gzip-based kernels (which approximate the chosen compressor/D2KE population kernel—not the ideal Solomonoff kernel, for which no general approximation theorem is supplied; see Section 8.2) and still trust the resulting MMD and HSIC values.

**Theorem** **23** (MMD Perturbation under Uniform Kernel Approximation)**.** *Let $k,\hat{k}$ be bounded measurable PSD kernels on X. Assume that*

(137) $$\underset{x,x^{\prime }\in X}{\sup}\left|k\left(x,x^{\prime }\right)-\hat{k}\left(x,x^{\prime }\right)\right|\leq \delta .$$

*Then, for all probability measures P,Q on X:*

(138) $$\left|{\mathrm{MMD}}_{k}^{2}\left(P,Q\right)-{\mathrm{MMD}}_{\hat{k}}^{2}\left(P,Q\right)\right|\leq 4\delta .$$

**Proof.**  Recall that ${\mathrm{MMD}}_{k}^{2}\left(P,Q\right)=E\left[k\left(X,X^{\prime }\right)\right]+E\left[k\left(Y,Y^{\prime }\right)\right]-2E\left[k\left(X,Y\right)\right]$, where $X,X^{\prime }∼P$ i.i.d. and $Y,Y^{\prime }∼Q$ i.i.d. Therefore,

(139) $$\begin{array}{cc} {\mathrm{MMD}}_{k}^{2}\left(P,Q\right)-{\mathrm{MMD}}_{\hat{k}}^{2}\left(P,Q\right)\; & =E[k\left(X,X^{\prime }\right)-\hat{k}\left(X,X^{\prime }\right)], \end{array}$$

(140) $$\begin{array}{cc} \; & \;+E[k\left(Y,Y^{\prime }\right)-\hat{k}\left(Y,Y^{\prime }\right)], \end{array}$$

(141) $$\begin{array}{cc} \; & \;-2E[k\left(X,Y\right)-\hat{k}\left(X,Y\right)]. \end{array}$$

Taking absolute values and applying the uniform bound yields $|{\mathrm{MMD}}_{k}^{2}-{\mathrm{MMD}}_{\hat{k}}^{2}|\leq \delta +\delta +2\delta =4\delta$.    □

**Proposition** **13** (Sample-local MMD perturbation certification)**.** *On pooled observations $z_{1:n+m}=\left(x_{1:n},y_{1:m}\right)$, let $w_{i}=1/n$ for i≤n and $w_{i}=-1/m$ otherwise, and let $G,\hat{G}$ be two evaluated Gram matrices. Their biased empirical discrepancies satisfy*

(142) $$\left|{\hat{\mathrm{MMD}}}_{b,G}^{2}-{\hat{\mathrm{MMD}}}_{b,\hat{G}}^{2}\right|=\left|w^{⊤}\left(G-\hat{G}\right)w\right|\leq \sum_{i,j}|w_{i}w_{j}\left|\;\right|G_{ij}-{\hat{G}}_{ij}|.$$

*Thus, heterogeneous, pair-specific approximation certification can be much tighter than four times a global supremum.*

**Proof.**  The biased MMD is the quadratic form $w^{⊤}Gw$. Subtract the two forms and apply the triangle inequality entrywise.    □

**Remark** **24** (Certification, not hyperparameter selection)**.** *Theorem 23 is a worst-case stability certification. Its global supremum can be very conservative, as the numerical ratio in Section 8.14 demonstrates, and it should not be presented as a practical rule for choosing γ or the landmark budget. The local bound (142) is useful only when the pairwise errors themselves are observable or certified. When the reference is the uncomputable ideal kernel, they are not. Data-dependent tuning must use independent training or validation data, or a label-invariant or correctly permutation-recomputed procedure that preserves the final test’s calibration.*

**Theorem** **24** (HSIC Perturbation under Uniform Kernel Approximation)**.** *Let $k,\hat{k}$ be kernels on X and $l,\hat{l}$ kernels on Y. Assume that*

(143) $$\underset{x,x^{\prime }}{\sup}|k\left(x,x^{\prime }\right)|\leq B_{k},\;\underset{x,x^{\prime }}{\sup}\left|k\left(x,x^{\prime }\right)-\hat{k}\left(x,x^{\prime }\right)\right|\leq \delta_{k},$$

(144) $$\underset{y,y^{\prime }}{\sup}|l\left(y,y^{\prime }\right)|\leq B_{l},\;\underset{y,y^{\prime }}{\sup}\left|l\left(y,y^{\prime }\right)-\hat{l}\left(y,y^{\prime }\right)\right|\leq \delta_{l}.$$

*Then, for every joint law $P_{XY}$:*

(145) $$\left|{\mathrm{HSIC}}_{k,l}\left(P_{XY}\right)-{\mathrm{HSIC}}_{\hat{k},\hat{l}}\left(P_{XY}\right)\right|\leq 4\left(B_{l}\delta_{k}+B_{k}\delta_{l}+\delta_{k}\delta_{l}\right).$$

**Proof.**  Let q=k⊗l and $\hat{q}=\hat{k}⊗\hat{l}$. Uniformly,

(146) $$|q-\hat{q}|\leq B_{k}\delta_{l}+\left(B_{l}+\delta_{l}\right)\delta_{k}=B_{l}\delta_{k}+B_{k}\delta_{l}+\delta_{k}\delta_{l}.$$

By Proposition 9, both HSIC quantities are squared MMDs between $P_{XY}$ and $P_{X}⊗P_{Y}$. Apply Theorem 23.    □

**Corollary** **5** (End-to-End Approximation Pipeline)**.** *If $\hat{k}$ is the truncated Solomonoff kernel $k_{F}$ from Theorem 14 with τ(F)≤δ, and l is any fixed bounded kernel with ${∥l∥}_{\infty }\leq B_{l}$, then*

(147) $$|{\mathrm{MMD}}_{k_{\mathrm{Sol}}}^{2}\left(P,Q\right)-{\mathrm{MMD}}_{k_{F}}^{2}\left(P,Q\right)|\leq 4\delta ,$$

(148) $$|{\mathrm{HSIC}}_{k_{\mathrm{Sol}},l}\left(P_{XY}\right)-{\mathrm{HSIC}}_{k_{F},l}\left(P_{XY}\right)|\leq 4B_{l}\delta .$$

*As F↑Ω, δ→0 and the truncated discrepancies converge to their ideal counterparts.*

**Remark** **25** (Significance)**.** *Theorems 23 and 24 show that a* given *uniform kernel approximation propagates linearly into MMD^2^ and the HSIC. The Solomonoff truncation result and the compressor joint-code result instantiate this premise in different, nonidentical settings. They do not jointly prove that a practical compressor converges to the Solomonoff kernel.*

## 7. Finite-Sample Theory for Both Directions

Section 6 controlled the gap between ideal objects and computable surrogates. A second gap remains, between the population discrepancies of Section 5 and what can be computed from finitely many samples and finitely many landmarks. This section bounds it. The two error sources are kept separate: landmark Monte Carlo error, which is controlled for landmarks drawn independently of the data, and sampling error, which is controlled for a fixed kernel. Combining them under data-dependent landmark selection is left open, and Section 8 says what that means for the procedures.

This section provides statistical guarantees for the estimators.

### 7.1. D2KE Approximation Quality

**Theorem** **25** (D2KE Multiplicative Bounds)**.** *Assume that d:X×Λ→[0,∞) satisfies the triangle-type inequality d(x,ω)+d(y,ω)≥d(x,y) for all x,y∈X and ω∈Λ, where d(x,y) is a dissimilarity on X. For a landmark set $S=\{\omega_{1},\ldots ,\omega_{m}\}$ with uniform weights:*

(149) $$k_{D2\mathrm{KE}}^{\left(m\right)}\left(x,y\right)=\frac{1}{m}\sum_{j=1}^{m}e^{-\gamma d(x,\omega_{j})}e^{-\gamma d(y,\omega_{j})}.$$

*Let $R_{x,y}=\max_{s\in S}\left\{d\left(x,s\right)\vee d\left(y,s\right)\right\}$. Then,*

(150) $$\frac{1}{m}e^{-2\gamma R_{x,y}}e^{-\gamma d(x,y)}\leq k_{D2\mathrm{KE}}^{\left(m\right)}\left(x,y\right)\leq e^{-\gamma d(x,y)}.$$

**Proof.**  **Upper bound:** By the triangle inequality, d(x,ω)+d(y,ω)≥d(x,y). Hence, each term $e^{-\gamma d(x,\omega )}e^{-\gamma d(y,\omega )}\leq e^{-\gamma d(x,y)}$. Averaging preserves the inequality.

**Lower bound:** Let $\omega^{*}\in S$ minimize d(x,ω)+d(y,ω) over *S*. Then,

(151) $$k^{\left(m\right)}\geq \frac{1}{m}e^{-\gamma d(x,\omega^{*})}e^{-\gamma d(y,\omega^{*})}.$$

Since $d\left(x,\omega^{*}\right),d\left(y,\omega^{*}\right)\leq R_{x,y}$, $d\left(x,\omega^{*}\right)+d\left(y,\omega^{*}\right)\leq 2R_{x,y}$. Therefore,

(152) $$e^{-\gamma d(x,\omega^{*})}e^{-\gamma d(y,\omega^{*})}=e^{-\gamma (d\left(x,\omega^{*}\right)+d\left(y,\omega^{*}\right))}\geq e^{-2\gamma R_{x,y}}\geq e^{-2\gamma R_{x,y}}e^{-\gamma d(x,y)},$$

where the last inequality uses only d(x,y)≥0. The triangle-type inequality is needed for the upper bound, not for this lower-bound step. This proves the lower bound.    □

### 7.2. D2KE Convergence

**Theorem** **26** (D2KE Kernel Convergence)**.** *Let M:=π(Λ)∈(0,∞) and $\bar{\pi }:=\pi /M$. For i.i.d. landmarks $\omega_{1},\ldots ,\omega_{m}∼\bar{\pi }$, define the empirical D2KE kernel by*

(153) $$k_{D2\mathrm{KE}}^{\left(m\right)}\left(x,y\right)=\frac{M}{m}\sum_{j=1}^{m}e^{-\gamma d(x,\omega_{j})}e^{-\gamma d(y,\omega_{j})},$$

*so that its expectation is the finite-measure kernel in Definition 18. Then,*

(154) $$P\left(|k_{D2\mathrm{KE}}^{\left(m\right)}\left(x,y\right)-k_{D2\mathrm{KE}}\left(x,y\right)|>\epsilon \right)\leq 2\exp\;\left(-\frac{2m\epsilon^{2}}{M^{2}}\right).$$

*For a probability landmark measure, M=1, and this reduces to the familiar unweighted empirical average and bound.*

**Proof.**  Define $Z_{j}=Me^{-\gamma d(x,\omega_{j})}e^{-\gamma d(y,\omega_{j})}$. The $Z_{j}$ are i.i.d., $Z_{j}\in \left[0,M\right]$, and 

(155) $$E_{\bar{\pi }}\left[Z_{j}\right]=M\int e^{-\gamma d(x,\omega )}e^{-\gamma d(y,\omega )}\;d\bar{\pi }\left(\omega \right)=k_{D2\mathrm{KE}}\left(x,y\right).$$

The empirical kernel is $k_{D2\mathrm{KE}}^{\left(m\right)}\left(x,y\right)=\frac{1}{m}\sum_{j=1}^{m}Z_{j}$.

By Hoeffding’s inequality for bounded random variables,

(156) $$P\left(\left|\frac{1}{m}\sum_{j=1}^{m}Z_{j}-E\left[Z_{1}\right]\right|>\epsilon \right)\leq 2\exp\;\left(-\frac{2m\epsilon^{2}}{M^{2}}\right).\;□$$

**Corollary** **6** (Uniform control on an observed Gram matrix)**.** *For fixed observations $z_{1},\ldots ,z_{N}$, the same landmarks and M=π(Λ) satisfy*

(157) $$\mathrm{Pr}\;\left(\max_{1\leq i,j\leq N}\left|k_{D2\mathrm{KE}}^{\left(m\right)}\left(z_{i},z_{j}\right)-k_{D2\mathrm{KE}}\left(z_{i},z_{j}\right)\right|>\epsilon \right)\leq 2N^{2}\exp\;\left(-\frac{2m\epsilon^{2}}{M^{2}}\right).$$

*This is a finite-set union bound; Theorem 26 alone is only pointwise and does not claim uniform convergence over an infinite domain. For a Markov KSD computed from finite transition supports, the finite evaluation set must also include every transition-support state at which the kernel is evaluated. For sampled transitions it must include the transition draws. A dataset-only event does not control those additional entries, and this finite-set certification cannot cover an infinite transition support without an additional tail or averaging bound.*

### 7.3. MMD Consistency

For independent samples $X_{1:n}∼P$ and $Y_{1:m}∼Q$, define the biased V-statistic

(158) $$\begin{array}{cc} {\hat{\mathrm{MMD}}}_{b,k}^{2}\; & :=\frac{1}{n^{2}}\sum_{i,j}k\left(X_{i},X_{j}\right)+\frac{1}{m^{2}}\sum_{i,j}k\left(Y_{i},Y_{j}\right)-\frac{2}{nm}\sum_{i,j}k\left(X_{i},Y_{j}\right) \end{array}$$

and the unbiased U-statistic

(159) $$\begin{array}{cc} {\hat{\mathrm{MMD}}}_{u,k}^{2}\; & :=\frac{1}{n(n-1)}\sum_{i\neq j}k\left(X_{i},X_{j}\right)+\frac{1}{m(m-1)}\sum_{i\neq j}k\left(Y_{i},Y_{j}\right)-\frac{2}{nm}\sum_{i,j}k\left(X_{i},Y_{j}\right). \end{array}$$

The V-statistic is non-negative for a PSD evaluated Gram matrix. The U-statistic is unbiased but may be negative in a finite sample. If 0≤k≤B, then

(160) $$\left|E{\hat{\mathrm{MMD}}}_{b,k}^{2}-{\mathrm{MMD}}_{k}^{2}\left(P,Q\right)\right|\leq B\left(\frac{1}{n}+\frac{1}{m}\right).$$

Only the diagonal terms contribute to this bias.

**Proposition** **14** (MMD Consistency for Bounded Kernels)**.** *Let k be a bounded measurable kernel with ${∥k∥}_{\infty }\leq B$. Then, the unbiased empirical MMD statistic based on independent samples from P and Q converges in probability to ${\mathrm{MMD}}^{2}\left(P,Q\right)$. Moreover, standard U-statistic bounds imply fluctuations of order $O_{p}\left(n^{-1/2}+m^{-1/2}\right)$.*

**Proof sketch.**  The unbiased MMD estimator is a U-statistic:

(161) $$\begin{array}{cc} {\hat{\mathrm{MMD}}}^{2}\; & =\frac{1}{n(n-1)}\sum_{i\neq j}k\left(X_{i},X_{j}\right)-\frac{2}{nm}\sum_{i,j}k\left(X_{i},Y_{j}\right),\\ \; & \;+\frac{1}{m(m-1)}\sum_{i\neq j}k\left(Y_{i},Y_{j}\right). \end{array}$$

Each term is unbiased for its population expectation. Consequently,

(162) $$E\left[{\hat{\mathrm{MMD}}}^{2}\right]={\mathrm{MMD}}^{2}\left(P,Q\right).$$

By Hoeffding’s decomposition for U-statistics, the variance of each term is O(1/n) or O(1/m) under boundedness. The total variance is therefore O(1/n+1/m), giving the stated rate.

For a complete proof with explicit constants, see [5] or standard references on U-statistics [39].    □

**Theorem** **27** (HSIC Consistency)**.** *For i.i.d. paired observations $\left(X_{i},Y_{i}\right)∼P_{XY}$ and fixed bounded KC-kernels k and l with ${∥k∥}_{\infty },{∥l∥}_{\infty }\leq B$:*

(163) $$\hat{\mathrm{HSIC}}\left({\hat{P}}_{XY,n}\right)\overset{p}{\to }\mathrm{HSIC}\left(P_{XY}\right)$$

*at rate $O_{p}\left(n^{-1/2}\right)$.*

**Proof.**  The unbiased HSIC estimator can be written as a U-statistic of order four:

(164) $$\hat{\mathrm{HSIC}}=\frac{1}{n(n-1)(n-2)(n-3)}\sum_{i\neq j\neq q\neq r}h\left(Z_{i},Z_{j},Z_{q},Z_{r}\right),$$

where $Z_{i}=\left(X_{i},Y_{i}\right)$ and *h* is a kernel function depending on *k* and *l* (the order-four *U*-statistic is due to [40]; Ref. [6] gives the biased trace estimator).

By the theory of U-statistics, this estimator is unbiased and its variance is O(1/n) under boundedness of the kernels. Hence, $\hat{\mathrm{HSIC}}\overset{p}{\to }\mathrm{HSIC}$ at rate $O_{p}\left(n^{-1/2}\right)$.    □

**Remark** **26** (Scope: continuous KSD)**.** *Continuous Langevin KSDs require differentiable densities, score functions, Stein classes, boundary conditions, and a careful choice between U- and V-statistic null limits. Those standard results are not needed for the countable-space Markov identity in Theorem 21; to keep the contribution focused, we do not restate them here.*

### 7.4. Concentration Inequality

**Theorem** **28** (Hoeffding-Type Bound for the Unbiased MMD Estimator)**.** *Let n,m≥2 and let 0≤k(x,y)≤B. For the unbiased U-statistic ${\hat{\mathrm{MMD}}}_{u,k}^{2}$ in Equation (159), with probability at least 1−δ,*

(165) $$|{\hat{\mathrm{MMD}}}_{u,k}^{2}-{\mathrm{MMD}}_{k}^{2}\left(P,Q\right)|\leq 2B\sqrt{2\left(\frac{1}{n}+\frac{1}{m}\right)\log\left(2/\delta \right)}\leq 2B\sqrt{\frac{2\log(2/\delta )}{n}}+2B\sqrt{\frac{2\log(2/\delta )}{m}}.$$

*The zero-bias centering in this statement does not apply to the biased V-statistic, whose bias is bounded separately in Equation (160).*

**Proof.**  View ${\hat{\mathrm{MMD}}}_{u,k}^{2}$ as a function of the n+m samples. Its expectation is ${\mathrm{MMD}}_{k}^{2}\left(P,Q\right)$ by the U-statistic construction.

*Bounded differences.* Changing one sample $X_{i}$ to $X_{i}^{\prime }$ changes the first sum by at most $\frac{2(n-1)}{n(n-1)}B=\frac{2B}{n}$ and the cross term by at most $\frac{2m}{nm}B=\frac{2B}{n}$. Thus, the total change is at most $\frac{4B}{n}$.

Similarly, changing $Y_{j}$ changes the estimate by at most $\frac{4B}{m}$.

**McDiarmid’s inequality:**

(166) $$P\left({\hat{\mathrm{MMD}}}_{u,k}^{2}-E\left[{\hat{\mathrm{MMD}}}_{u,k}^{2}\right]>t\right)\leq \exp\left(-\frac{2t^{2}}{n{\left(4B/n\right)}^{2}+m{\left(4B/m\right)}^{2}}\right)=\exp\left(-\frac{t^{2}}{8B^{2}\left(1/n+1/m\right)}\right).$$

Setting the right side to δ/2 and solving gives

(167) $$t=2B\sqrt{2(1/n+1/m)\log(2/\delta )}.$$

A union bound over upper and lower tails gives the first inequality in (165). The displayed decoupled form follows from $\sqrt{a+b}\leq \sqrt{a}+\sqrt{b}$ for a,b≥0.    □

**Remark** **27.** *The range assumption 0≤k≤B is used in the bounded-difference constants. If only |k|≤B is known, replacing one kernel value can change it by 2B, and the displayed constants should be doubled.*

## 8. Practice: Protocols, Numerical Evidence, Limitations, and Open Problems

The preceding sections separated what is proved at the ideal layer (Section 4 and Section 5), what survives the passage to computable surrogates (Section 6), and what can be estimated from finitely many samples (Section 7). This section closes the loop. It records the status of every interface established above, states the three procedures those interfaces license, reports the numerical evidence for them, delimits what none of it establishes, and ends with the questions left open.

### 8.1. Status of the Interfaces

Table 3 collects the status of every interface established above, together with the hypothesis each one cannot do without.

### 8.2. Limitations

We explicitly state the limitations of this work.

### 8.3. Computability

The main limitation is computability. The most natural objects from the AIT side (Kolmogorov complexity, Solomonoff prior, ideal Solomonoff kernel) are uncomputable. The paper proves exact statements for these ideal objects and uses them to motivate practical surrogates, but it does not supply a general approximation theorem from compressor-based kernels to Solomonoff- or Kolmogorov-based kernels.

Any empirical success of gzip-, LZMA-, or neural-compressor-based methods should be viewed as evidence for the usefulness of the compression-to-kernel paradigm, not as a theorem about universality.

### 8.4. Characteristicity

Positive semidefiniteness does not imply characteristicity. For the Solomonoff kernel, Proposition 4 gives the exact signed-measure criterion, but no machine-independent guarantee holds: Theorem 13 gives failure for $U^{-}$ and characteristicity on all countable probability laws for $U^{+}$. The pair-complexity equivalence alone is insufficient. For a practical kernel, finite-support characteristicity certifies separation of all laws on that support; it is neither necessary for power against a particular alternative nor sufficient for finite-sample power.

### 8.5. Independence and AMI

Theorem 17 is an expectation theorem for a fixed computable source description. It does not imply that every individual draw from independent sources has small algorithmic mutual information, nor does it identify the HSIC numerically with AMI.

### 8.6. No Practical Compressor Guarantees

We do not establish that any specific compressor (gzip, bzip2, LZMA, etc.) achieves a particular approximation error ε(n) on any particular class $C_{n}$. Such guarantees would require a separate analysis for each compressor family.

### 8.7. Statistical Compressors Versus Direct Algorithmic-Probability Estimation

The empirical layer of this paper relies on off-the-shelf lossless compressors (gzip, zlib, LZMA), which approximate entropy-rate and repeated-pattern statistics rather than algorithmic complexity proper. This is a limitation of the surrogate, not of the ideal objects: every discrepancy statistic studied here consumes code lengths only through the kernel, so any computable surrogate can be substituted. In particular, the coding theorem method (CTM) and block decomposition method (BDM) of Zenil, Soler-Toscano, Delahaye and collaborators [41,42,43] estimate algorithmic probability directly by small-machine enumeration, and are the natural surrogates in the short-string regime where LZ-family compressors saturate; CTM/BDM-instantiated kernels are drop-in replacements in the MMD/HSIC/KSD constructions of this paper.

### 8.8. Three Computational Protocols

We present three practical algorithms implementing the theoretical bridges. All rely on a real-world compressor *C* (e.g., gzip, LZMA) and the D2KE construction. These protocols operate at Layer B/C: they use computable compressor surrogates and finite-sample estimators.

### 8.9. Finite-Sample Validity of the Randomization Tests

The finite-sample point-estimation theory of Section 7 is proved for landmarks drawn independently of the evaluation data. Protocols 1–2 therefore distinguish an external or held-out landmark design, to which those bounds apply, from a pooled permutation-equivariant design. The latter still has exact randomization validity but does *not* inherit the $O(n^{-1/2}+m^{-1/2})$ point-estimation bounds: changing one observation that also serves as a landmark can perturb every feature row. The joint theory for that case is Open Problem O5.

**Definition** **23** (Permutation-equivariant landmark rule)**.** *Let $Z=(z_{1},\ldots ,z_{n+m})$ be the pooled sample. A landmark rule S=S(Z) is* permutation-equivariant *if S(σZ)=S(Z) for every permutation σ of the indices, i.e., S depends on Z only through the unordered multiset.*

**Proposition** **15** (Exact conditional level)**.** *Suppose S is permutation-equivariant and the features $\phi_{s}\left(\cdot \right)$, s∈S are computed from S alone. Then, under $H_{0}:P=Q$, for every B≥1, every |S|, and every γ>0, the randomization p-value $p=\left(1+\#\left\{b:V_{b}\geq V\right\}\right)/\left(B+1\right)$, using independent uniform permutations, satisfies*

(168) Pr(p≤α∣M)≤αforeveryα∈(0,1).

*Here, M is the unordered pooled multiset, not the ordered tuple Z. The same holds for Protocol 2 under $H_{0}:X⊥Y$, conditional on the two unordered marginal multisets, with independent uniform permutations of the Y-indices.*

**Proof.**  Under $H_{0}$, the pooled sample is exchangeable, so conditionally, on the unordered multiset M, the ordered sample is uniform on the orbit of the symmetric group. Equivariance makes *S*, hence the feature map, and hence the map from a labeling to the statistic, a fixed measurable function on that orbit. The statistic and its *B* randomization replicates are therefore exchangeable, and the plus-one randomization *p*-value of a set of B+1 exchangeable values is super-uniform [44]. Nothing in the argument uses the distribution of *S*, so the level does not depend on |S| or γ. The HSIC case is identical with the group of *Y*-index permutations, under which $S_{X}$ and $S_{Y}$ are fixed by construction.    □

**Remark** **28** (What this does and does not give)**.** *Proposition 15 controls the type I error exactly, for any landmark budget and bandwidth. It says nothing about power, which does depend on |S| and γ, and nothing about the accuracy of V as an estimate of the population discrepancy. A rule such as “take every other element of the pooled list” is* not *equivariant—it stratifies by label—and voids the guarantee. For fixed kernels, or conditionally on an independently chosen external landmark set held fixed as sample sizes grow, the U-statistics are first-order degenerate under $H_{0}$. Their scaled null limits are weighted $\chi^{2}$ mixtures $\sum_{j}\lambda_{j}\left(Z_{j}^{2}-1\right)$ rather than Gaussian. For two-sample MMDs with comparable sample sizes n≍m, and for the HSIC with n paired observations, the null rate is $O_{p}\left(n^{-1}\right)$. The weaker consistency bounds in Proposition 14 and Theorem 27 remain valid but need not be sharp under the null. These fixed-kernel asymptotics, and the associated conditions for a multiplier bootstrap, are not established here for adaptive pooled landmarks.*

**Remark** **29** (Why Protocol 3 needs a different calibration)**.** *The KSD null distribution depends on P through $T_{P}$, so label permutation is not available. Step 5 calibrates by exact simulation from P. This is a real restriction: if exact simulation from P is available, a permutation MMD test between the sample and simulated draws is also available, is exactly calibrated by Proposition 15, and requires no choice of $T_{P}$. The exact calibration in Step 5 costs Bn target draws, whereas a two-sample MMD permutation test needs only one target sample of size n and reuses it across all permutations. Protocol 3 therefore has no simulation-cost advantage under this exact calibration. Its reasons for use are instead operator-based diagnostics, reuse of a single calibrated null distribution across candidate samples of the same size, or settings in which local $T_{P}$-averages are scientifically meaningful. A multiplier bootstrap can avoid repeated target simulation only asymptotically and under the usual degenerate-U-statistic conditions. We do not claim that KSD dominates a two-sample test. The detection theorem requires that P be the unique stationary law; irreducibility is a sufficient condition on a finite state space, rather than an additional necessary hypothesis of that theorem.*

*Discrepancy magnitude and calibrated power must be distinguished. For a fixed target-stationary $T_{0}$, put $T_{\varepsilon }=\left(1-\varepsilon \right)I+\varepsilon T_{0}$, where 0<ε≤1. Direct substitution gives*

$$\xi_{T_{\varepsilon }}=\varepsilon \xi_{T_{0}},\;h_{T_{\varepsilon }}=\varepsilon^{2}h_{T_{0}}.$$

*Both the observed and every simulated-null statistic in Protocol 3 are multiplied by $\varepsilon^{2}$. Their rank comparisons and the resulting p-values are therefore identical for every ε>0, even though the population KSD tends to zero as ε↓0. At ε=0, every statistic is zero. Operator geometry can affect power, but positive lazy rescaling alone does not reduce the power of this exactly calibrated test.*

#### 8.9.1. Protocol 1: Algorithmic MMD Two-Sample Test

Algorithm 1 implements the two-sample test; Algorithms 2 and 3 implement the independence and model-criticism tests.

#### 8.9.2. Protocol 2: Algorithmic HSIC Independence Test

We test independence using the centered marginal Gram matrices.

#### 8.9.3. Protocol 3: Discrete Algorithmic KSD Model Criticism

The transition-feature residual defines the simple-null goodness-of-fit test.
**Algorithm 1** Algorithmic MMD Two-Sample Test**Require:** Independent i.i.d. samples ${\left\{x_{i}\right\}}_{i=1}^{n}∼P$, ${\left\{y_{j}\right\}}_{j=1}^{m}∼Q$, compressor *C*, bandwidth γ, landmark budget *r*, randomization budget *B*

**Ensure:** Non-negative statistic and randomization *p*-value for $H_{0}:P=Q$
1:Select *r* landmarks from an external corpus or a held-out split if Section 7’s estimation bounds are required. Otherwise, a pooled rule must be permutation-equivariant (e.g., the *r* lexicographically smallest distinct pooled strings), which preserves only the randomization guarantee.2:For every observation–landmark pair (z,s), compute $\ell_{C}\left(z\right)$, $\ell_{C}\left(s\right)$, and $\ell_{C}^{\mathrm{pair}}\left(z,s\right)$, then set $d_{C}\left(z,s\right)=\max\left\{0,{\mathrm{NCD}}_{C}\left(z,s\right)\right\}$ using Definition 8.3:Form the feature vector $\Phi \left(z\right)={\left|S\right|}^{-1/2}{\left(e^{-\gamma d_{C}\left(z,s\right)}\right)}_{s\in S}$ and kernel $k\left(z,z^{\prime }\right)=\langle \Phi \left(z\right),\Phi \left(z^{\prime }\right)\rangle$.4:Compute the biased statistic $V=∥\;n^{-1}\sum_{i}\Phi \left(x_{i}\right)-m^{-1}\sum_{j}\Phi \left(y_{j}\right){∥}_{2}^{2}$.5:For b=1,…,B, permute the pooled labels and recompute $V_{b}$ with the same features6:Set $p=\left(1+\#\left\{b:V_{b}\geq V\right\}\right)/\left(B+1\right)$7:**return** V,p


**Algorithm 2** Algorithmic HSIC Independence Test**Require:** I.i.d. paired observations ${\left\{\left(x_{i},y_{i}\right)\right\}}_{i=1}^{n}$, compressor *C*, bandwidth γ, marginal landmark budgets $r_{X},r_{Y}$, randomization budget *B*

**Ensure:** Compression-kernel dependence statistic and permutation *p*-value
1:Select external or held-out $S_{X},S_{Y}$ for the estimation bounds. If marginal observations are used, the rules must depend only on the two marginal multisets, not on the pairing (Definition 23).2:Build D2KE matrices $G_{k}=\Phi_{X}\Phi_{X}^{⊤}$ and $G_{l}=\Phi_{Y}\Phi_{Y}^{⊤}$ from marginal non-negative NCD features, as in Protocol 13:Center: ${\tilde{G}}_{k}=\Pi G_{k}\Pi$, ${\tilde{G}}_{l}=\Pi G_{l}\Pi$, where $\Pi =I-\frac{1}{n}11^{⊤}$4:Compute biased $V=n^{-2}\mathrm{tr}\left({\tilde{G}}_{k}{\tilde{G}}_{l}\right)$5:Permute the *Y* indices *B* times and set $p=\left(1+\#\left\{b:V_{b}\geq V\right\}\right)/\left(B+1\right)$6:**return** V,p (a dependence statistic, not an estimator of AMI)


**Algorithm 3** Discrete Model Criticism via KSD**Require:** I.i.d. observations ${\left\{x_{i}\right\}}_{i=1}^{n}∼Q$, exact i.i.d. sampling from *P*, a *P*-stationary kernel $T_{P}$ with finitely enumerable transition supports and evaluable probabilities (or exactly evaluable feature averages $T_{P}\Phi$), compressor *C*, bandwidth γ, landmark budget *r*, Monte Carlo budget *B*

**Ensure:** Test statistic and exact simple-null Monte Carlo *p*-value for $H_{0}:Q=P$
1:Draw *r* landmarks *S from P*, or from a sample split held out from $\left\{x_{i}\right\}$, so that *S* is independent of the evaluation sample; construct finite D2KE features Φ using non-negative NCD dissimilarities.2:Cache Φ on the observations and, for finite transition supports, the states in their one-step supports.3:Evaluate $\Psi \left(x_{i}\right)=\left(T_{P}\Phi \right)\left(x_{i}\right)-\Phi \left(x_{i}\right)$ by the finite sum or the supplied exact averaging procedure.4:Compute the biased statistic $V=∥n^{-1}\sum_{i}\Psi \left(x_{i}\right){∥}_{2}^{2}$5:For b=1,…,B, generate an independent i.i.d. sample of size *n* from *P* and recompute $V_{b}$ using the same features and averaging procedure, expanding the finite cache as needed.6:Set $p=\left(1+\#\left\{b:V_{b}\geq V\right\}\right)/\left(B+1\right)$7:**return** V,p

**Remark** **30.** *These protocols are motivated by the ideal theory but do not inherit its guarantees automatically. Theorem 22 controls the Layer-B/A gap only under its explicit finite-family approximation hypothesis. Type I error for Protocols 1 and 2 is controlled by Proposition 15. Protocol 3 instead uses the exchangeability of the observed and exactly simulated null statistics, conditional on the independently selected landmarks. The target P and operator $T_{P}$ must also be fixed in advance or selected independently of the evaluation observations. Fitting either from the evaluation data gives a composite or adaptive null problem requiring separate calibration. Its implementation requires the sampling and averaging capabilities stated in the algorithm; countability alone does not provide them. Consecutive observations from a stationary trajectory are generally dependent and do not satisfy the i.i.d. evaluation assumption; their calibration requires a separate dependent-data analysis. Distinct physical transition laws can also have the same stationary law, so none of these marginal-law tests identify transition dynamics.* No *result in Section 7 bounds the estimation error of V when the landmarks are drawn from the evaluation sample, because McDiarmid’s bounded-difference constant is then $O(∥k{∥}_{\infty })$ rather than $O(∥k{∥}_{\infty }/n)$: replacing one observation that is itself a landmark perturbs every entry of the Gram matrix. Use a held-out split if the point estimate, and not only the test decision, is to be interpreted. Ordinary label permutation is valid for the two-sample and independence nulls; it is not valid for the KSD, whose null distribution depends on P. Exact target simulation gives an exact simple-null Monte Carlo calibration. If exact target simulation is unavailable, a multiplier bootstrap may be used only under the usual degenerate-U-statistic conditions; a naive permutation between observations and paired one-step draws is invalid. The plus-one correction prevents impossible zero Monte Carlo p-values [44].*

### 8.10. Numerical Study

The following corrected study is a finite, synthetic demonstration of the identities, implementation conditions, and limitations above. It replaces the earlier numerical study after an audit identified a byte-encoding error, an incorrect Gram matrix in the resolution diagnostic, and inadequate negative controls. None of these experiments establish characteristicity on all strings or superiority on application data.

All compressor experiments use the non-negative dissimilarity $d_{C}\left(x,y\right)=\max\{0,$${\mathrm{NCD}}_{C}\left(x,y\right)\}$ without upper clipping, the symmetric length-prefixed pair code, and γ=1. Features are $\Phi_{i\ell }=\exp\left[-d_{C}\left(x_{i},w_{\ell }\right)\right]/\sqrt{m}$ for *m* uniformly weighted landmarks, and the D2KE Gram matrix is $G=\Phi \Phi^{⊤}$. The affinity matrix ${\left[\exp\left(-d_{C}\left(x_{i},x_{j}\right)\right)\right]}_{ij}$ is not substituted for *G*. Reported intervals are pointwise 95% Wilson binomial intervals computed from exact rejection counts. Synthetic replicate uncertainty is distinct from sensitivity to compressor, data family, or landmark choice.

### 8.11. Exact Finite Identities

For the profile identity, two samples of 20 strings and 36 landmarks give a Gram/profile difference of $1.04\times 10^{-16}$. Across 45 finite Markov configurations, the largest absolute difference between the Stein-feature, embedding-difference, and four-term kernel routes is $5.14\times 10^{-18}$. The largest stationarity residual is $1.67\times 10^{-16}$; at the stationary law, the largest feature energy is $3.39\times 10^{-33}$ and the largest absolute signed kernel energy is $8.76\times 10^{-20}$. These are floating-point identity checks, not statistical power evidence.

### 8.12. Finite Resolution and Characteristicity

**Figure 4:**
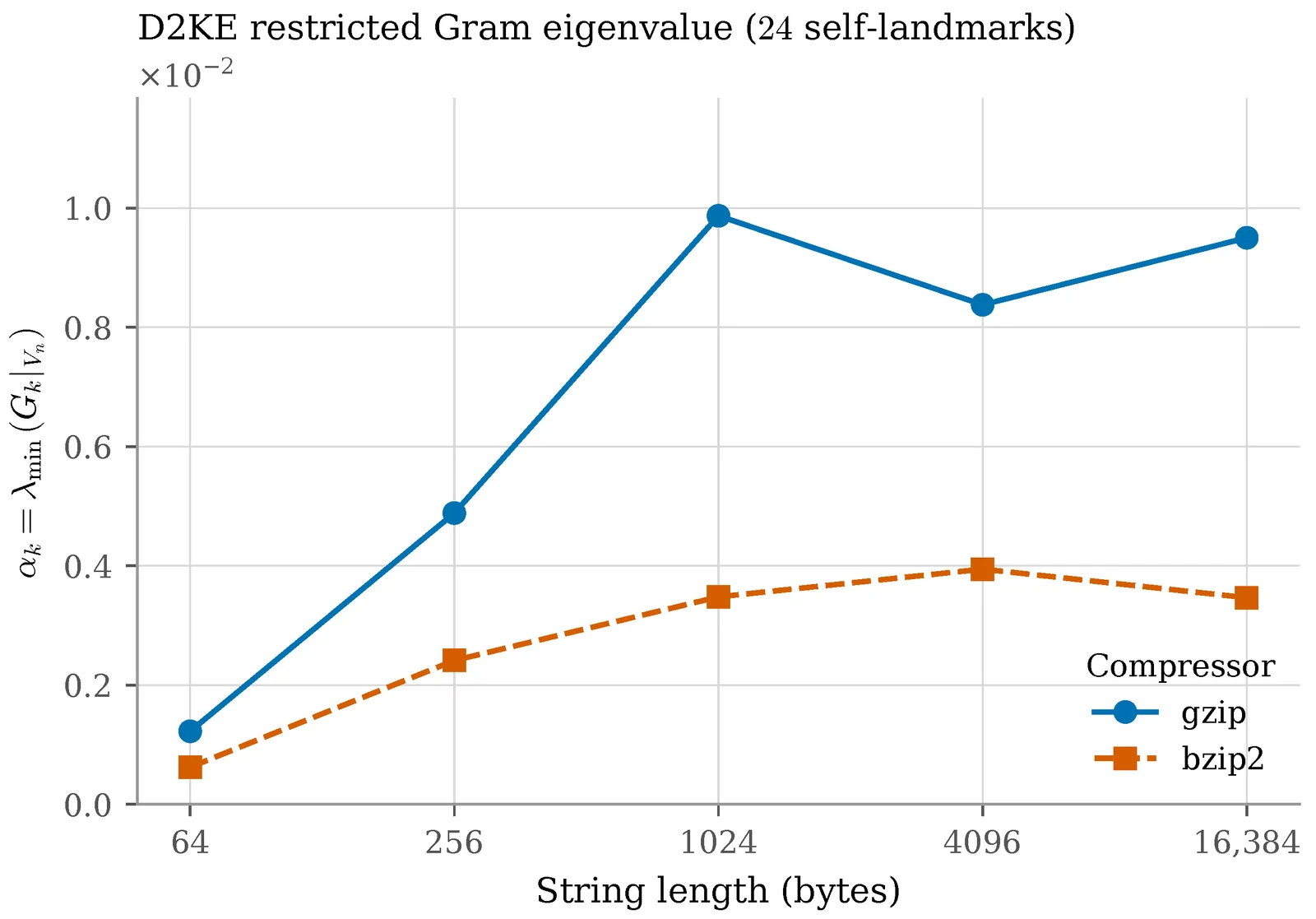
All ten restricted eigenvalues from Table 4. Each curve concerns finite self-landmark supports, not a global characteristicity claim.

At each length and compressor, the fixed seed-5 dataset contains eight iid, eight order-one Markov, and eight doubled strings. All 24 strings are used as self-landmarks. Table 4 reports distinct dissimilarities among 276 unordered off-diagonal pairs, the participation-ratio effective rank ${\left(\mathrm{tr}G\right)}^{2}/\mathrm{tr}\left(G^{2}\right)$, and the minimum eigenvalue of $U^{⊤}GU$, where *U* has orthonormal columns spanning the zero-sum subspace. Positive restricted eigenvalues certify separation on these particular finite supports up to numerical precision; they provide no uniform margin on strings. The length range is a factor of 256. Figure 4 plots the restricted eigenvalues.

### 8.13. A Controlled Two-Sample Comparison

The periodic generator draws a block of 600 actual uint8 symbols uniformly from {20,80,140,200}, then repeats and truncates it to 2400 bytes. Its alternative is a random-adjacency Hierholzer traversal of the directed five-mer multigraph, with the original endpoints retained. This shuffle always returns a string, preserves every linear *j*-mer count for 1≤j≤5, and is not uniform over all strings with these counts. Every sampled parent is retained; there are no rejected trials or substitute data families. Under the null, both samples are independent shuffled parents; under the alternative, one sample is periodic and the other is independently generated and shuffled. Related count-preserving randomization methods for nucleotide sequences were developed by Altschul and Erickson [45].

The RBF baselines use normalized *j*-mer count vectors hashed by CRC32 into 8192 buckets, with the squared-distance scale equal to the median pooled squared distance above $10^{-15}$ (scale one if none exists). They therefore have the same feature distribution under the two populations for j≤5; this does not mean that two independently drawn samples have identical histograms. The j=6 baseline is not constrained to be blind. D2KE uses the lexicographically smallest eight distinct pooled strings, with the actual landmark count in the normalization. Landmark selection and RBF scale are label-invariant.

**Figure 5:**
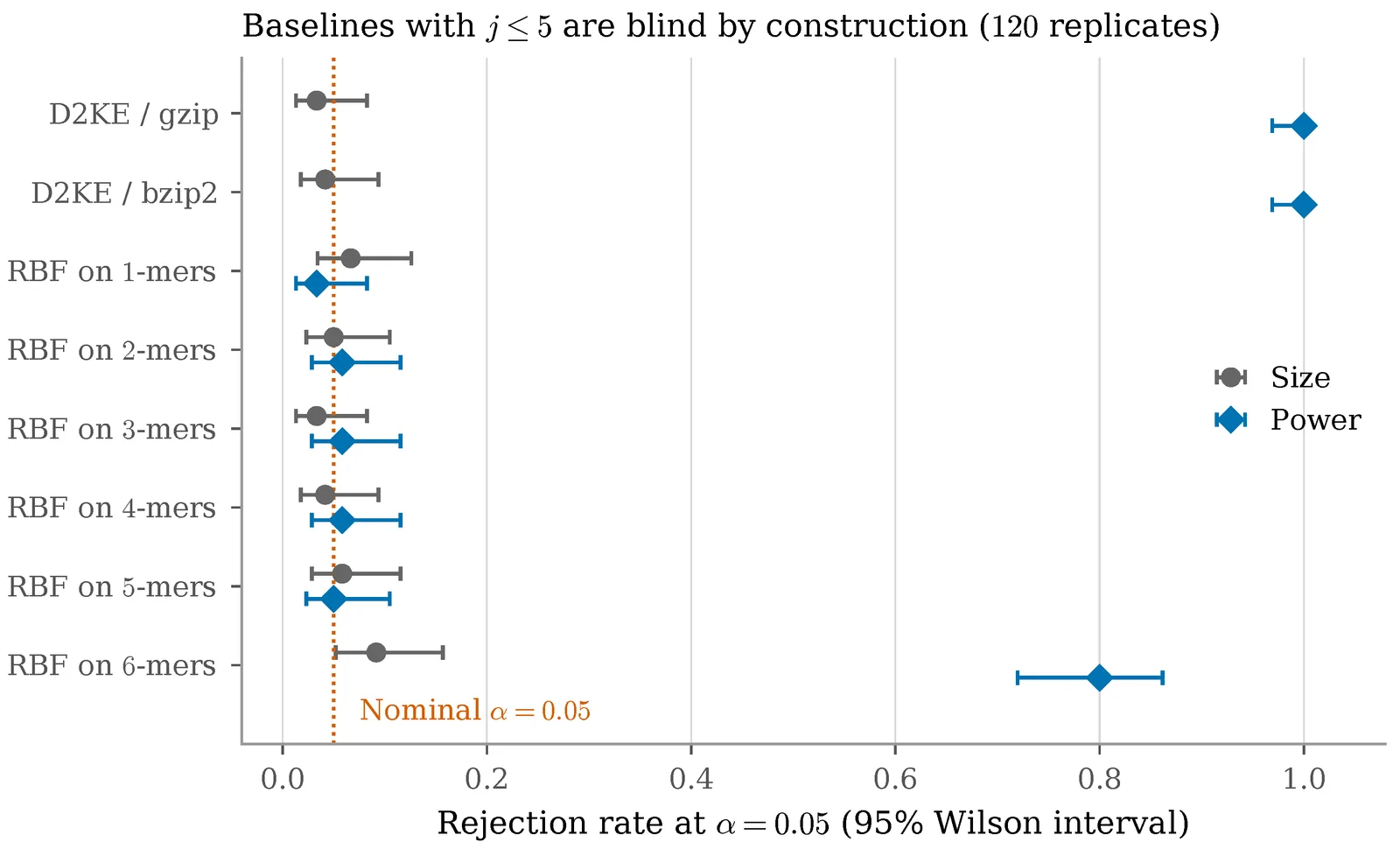
Table 5 with Wilson intervals. The five count-preserving baselines test a specific blind spot; the experiment is not an application benchmark.

Each arm has n=8 strings, each test uses B=199 random label permutations and the plus-one weak-tie *p*-value, and there are 120 replicates per hypothesis (seed 31). The independent construction check validates all five preserved count orders on ten additional parents. The experiment is deliberately diagnostic: the guaranteed blindness of five baselines is not evidence of broad competitiveness against tuned sequence kernels. Table 5 and Figure 5 report the rejection rates.

### 8.14. Landmark Sampling and Perturbations

For a fixed pair of length-1200 strings, a seed-77 pool comprises 60 periodic, 60 five-mer shuffled, 60 iid, and 60 Markov strings. For each budget, 400 samples drawn independently with replacement estimate the exact mean feature product of this fixed pool. Bounded feature products give the Hoeffding envelope

$$\sqrt{\log(2/0.05)/(2m)}.$$

It bounds each draw’s error probability; it need not bound all draws simultaneously.

**Figure 6:**
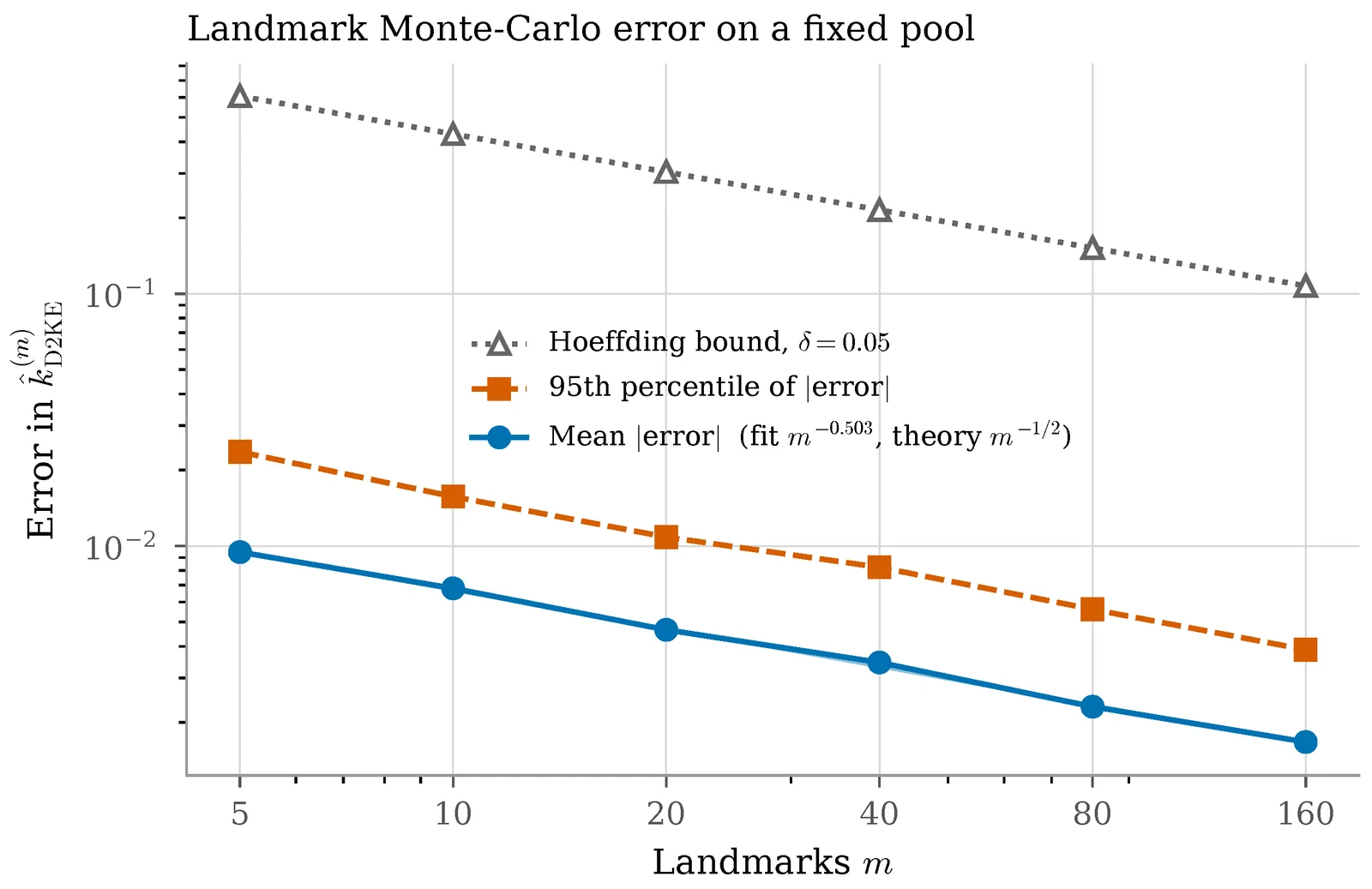
Table 6 on logarithmic axes. The thin line is a fitted-slope guide anchored at the first mean, not an additional experiment.

The feature-product variance is $6.91\times 10^{-4}$, and the log–log least-squares fit to the six mean errors has exponent −0.503. This one-pair diagnostic is compatible with Monte Carlo concentration, but does not estimate a distribution-wide convergence constant or optimize landmarks. Table 6 and Figure 6 report the sampling errors.

A separate 24-string, 20-landmark comparison of gzip and bzip2 profiles has ε=0.150476. The measured maximum kernel difference is $3.53\times 10^{-2}$, versus 2ε=0.300952; the squared-MMD difference is $1.81\times 10^{-3}$, versus 8ε=1.203807. These are finite compressor-to-compressor perturbations; neither compressor supplies ground-truth Kolmogorov complexity.

### 8.15. Finite Markov and Independence Diagnostics

The seed-101 Markov example has 8 states, 24 landmarks, a strictly positive row-stochastic transition matrix, and restricted margin $3.18\times 10^{-6}$. The stationary law is computed numerically. We draw 4000 candidate laws from a mixture of symmetric Dirichlet distributions with concentrations 0.4, 1, and 4, and bin them by distance from that law. Table 7 gives sample minima and medians, not global detection floors. The final bin contains the entire tail through distance two; sampled minima are not required to increase monotonically.

The main HSIC demonstration uses 20 pairs of length-400 strings and four latent types: periodic, four-mer shuffled, iid, and Markov. With probability ρ, the two strings share a latent type; otherwise, the second type is independently uniform. Independent strings are drawn conditional on their types. Thus, ρ=0 is independence, while ρ=1 shares a type, not an identical string. Each margin uses its eight lexicographically first distinct strings as landmarks, so its Gram matrix is equivariant under row permutation. There are 50 replicates per point, 199 permutations, and seed 5. The intervals quantify the small replicate budget.

**Figure 7:**
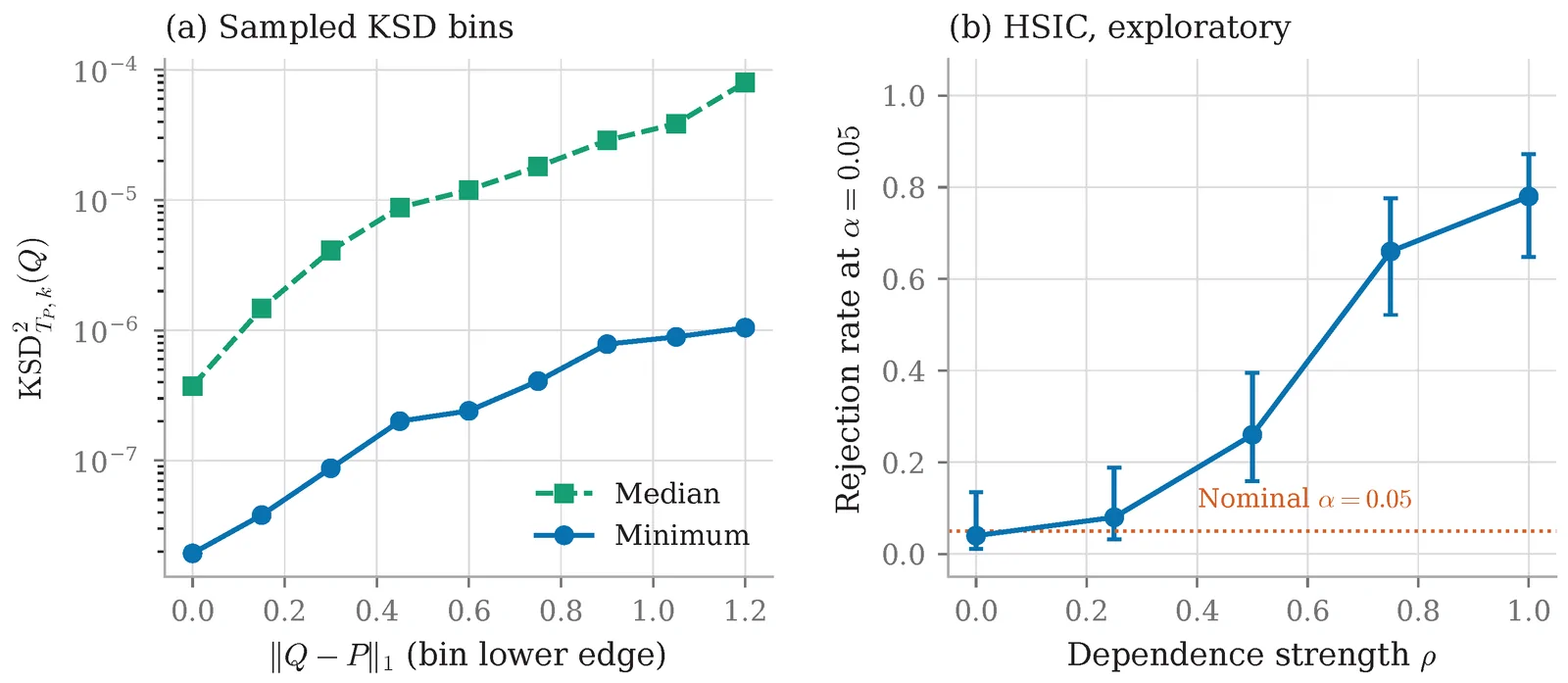
(**a**) All populated intervals from Table 7, shown at their lower edges. (**b**) Table 9, with pointwise Wilson intervals. Connecting lines are visual guides.

A separate reversal experiment (seed 101) uses 60 replicates per point, 12 pairs of length-600 strings, period 300, and six marginal landmarks. With probability ρ, *Y* is the byte reversal of *X*; otherwise, it is an independent periodic string. Table 8 reports this negative result separately. Strong dependence need not be visible to the selected compression features at this sample size. Neither demonstration compares the HSIC with specialized independence tests or establishes general power advantages. Table 9 reports the latent-type results. Figure 7 plots the KSD bins and latent-type HSIC results.

### 8.16. Controls and an Algebraic Power Boundary

The two KSD controls isolate different assumptions. A deficient feature map admits a nonstationary alternative invisible to the Stein features; a separating feature map still cannot distinguish distinct stationary laws of a reducible transition matrix.

The first control has d=6, m=2, ${∥Q-P∥}_{1}=0.217511$, and feature energy $6.79\times 10^{-36}$. The reducible control uses six distinct states as six self-landmarks: its Gram rank is 6, zero-sum rank is 5, and margin is $6.46\times 10^{-4}$. Its two closed classes yield distinct stationary laws with feature energies $1.39\times 10^{-33}$ and $3.09\times 10^{-33}$. The stationary probability simplex has an affine dimension of one. Signed kernel-route residuals near zero are numerical roundoff, not negative discrepancies.

For an exact power boundary, take four distinct states, delta dissimilarity, four uniform self-landmarks, and γ=log2 (the explicit exception to the compressor experiments’ bandwidth). Then, the D2KE Gram matrix is $G=\left(1/16\right)I+\left(3/8\right)11^{⊤}$, with positive restricted margin 1/16. Each balanced two-versus-two labeling has a squared MMD 1/16. Exhaustive enumeration of all six label subsets yields zero statistic spread and weak-tie randomization p=1 for every labeling. This is an algebraic counterexample to inferring finite-sample power from a positive characteristicity margin alone; it is not a 300-replicate compressor-data experiment.

### 8.17. Scope of the Evidence

Table 10 summarizes the scope of the evidence. The experiments do not validate the machine-dependent ideal Layer-A claims, universal characteristicity of a practical compressor kernel, scalable dynamical inference, or performance on real data. Within those limits, all plotted empirical quantities are tabulated above and independently checked from saved numerical inputs. Machine-readable inputs and exact counts, rather than rounded table entries, define the reported results.

### 8.18. Open Problems

O1. *Machine-invariant Solomonoff characteristicity.* Theorem 13 shows that Equation (62) is machine-dependent. Identify a natural subclass of universal prefix machines, or an additional normalization, for which the criterion holds uniformly over the subclass, and characterize the machines on each side.
O2. *Certified computable program mixtures.* Design computable program-feature kernels with observable tail certification and a meaningful restricted universality property.
O3. *Compressor approximation.* Obtain nonvacuous, domain-specific rates comparing named compressor families with prefix complexity or with a specified computable source class. Uniform all-string approximation is not expected.
O4. *Growing-alphabet AMI–HSIC theory.* Extend Theorem 18 to controlled growing supports while tracking restricted product-kernel eigenvalues and marginal masses.
O5. *Joint landmark and sampling theory.* Derive sharp finite-sample MMD, HSIC, and Markov-KSD bounds that simultaneously account for data, random landmarks, and data-dependent tuning without relying on sample splitting.
O6. *Markov-KSD power.* Quantify the power of ${\mathrm{MMD}}_{k}\left(QT_{P},Q\right)$ through the spectra of $T_{P}-I$ and the kernel integral operator, distinguishing changes in operator geometry from positive scalar rescaling, which leaves rank-calibrated power unchanged (Remark 29).
O7. *Normalized Solomonoff geometry.* Finite-support machine dependence already follows from diagonal rescaling and the two constructions (Remark 11). For the normalized kernel ${\bar{k}}_{\mathrm{Sol}}$ of Corollary 4, characterize when normalization preserves identification on all countable probability laws, including for $U^{+}$. Determine also which conditioning properties follow from normalization and how they relate to the pair-complexity interpretation.

## 9. Conclusions

The interface between algorithmic information and kernel discrepancies is a sequence of typed transformations, not an identification of Kolmogorov complexity with a test statistic. The two directions of Section 4 and Section 5 are not equivalent, and the difference between them is the main structural finding.

Going from algorithmic information to kernels, the constructions are unconditional. Compression dissimilarities enter through explicit features, feature inner products supply positive semidefiniteness, and zero-mass signed measures give the exact identification criterion, with finite matrix certification when the support is finite. This is not a general decision algorithm for ideal kernels. At the ideal end of this direction, the Solomonoff feature mixture is a bounded PSD kernel whose raw scale is

(169) $$k_{\mathrm{Sol},U}\left(x,y\right){≍}_{U}2^{-K_{U}\left(\langle x,y\rangle \right)},$$

so its logarithmic scale is joint prefix complexity up to a machine-dependent constant. The kernel is uncomputable, and we prove no general compressor approximation theorem for it.

Going the other way, the identities are exact but the algorithmic content thins. The MMD is the $L^{2}$ distance between compressibility profiles, the HSIC is the product-kernel MMD, and the Markov–Stein discrepancy is the pushforward MMD of Theorem 21: three clean statements about whatever kernel is supplied, none of which require the kernel to be algorithmic. The one genuinely algorithmic bridge, from expected algorithmic mutual information to the HSIC, holds only under source, support, and spectral conditions we make explicit, and its two-sided form has a constant we can name but not bound away from zero in general.

The reproducible synthetic experiments examine selected computable kernels, identities, approximation errors, and testing alternatives. Their conclusions are restricted to those constructions and sampling budgets; they establish neither a result about ideal AIT objects nor general superiority over competing features. The algebraic permutation-invariance example makes a separate point: a positive restricted eigenvalue certifies identification on the finite support, but does not guarantee power for the chosen finite-sample test. Conversely, a noncharacteristic kernel can detect alternatives outside its null space. Restricted eigenvalues and permutation dispersion therefore answer different questions, and empirical near-symmetry alone does not prove exact invariance.

## Figures and Tables

**Table 1:** Positioning. Established ingredients and the additional interface questions treated here.

Approach	Established Contribution	Additional Question Here	Treatment Here
Information distance; practical NCD	Ideal AIT distance; compressor-relative similarity on raw objects	Valid kernels and the assumptions linking practical code lengths to *K*	D2KE route plus the layer separation of Section 6
MMD, HSIC, KSD	Finite-sample theory, exact tests, detection under characteristic kernels	Identification for the selected compression features and explicit dependence on a countable-space Stein operator	Theorems 10, 11, 21
D2KE [2]	PSD features, landmark choices, metric regularity, and learning bounds	Distributional identification and discrepancy interpretation for compression features	Zero-mass criterion and the profile identity, Theorem 19
Parts I–VI	MDL kernels, spectral and learning-rate theory, the Solomonoff probabilistic layer	The discrepancy layer	This paper

**Table 2:** PSD realizations of KC geometry. Only D2KE and the Solomonoff feature kernel are used in the main discrepancy theorems.

Construction	Assumption	Status	Separation Condition
D2KE	Finite landmark measure and measurable *d*	Intrinsic	Theorem 11
Solomonoff	Fixed universal prefix machine	Ideal/intrinsic	Conditional
Exponential	*d* conditionally of negative type	Intrinsic if valid	Must be proved
MDS	Finite dataset	Sample-dependent	Affine rank check
Diffusion	Finite weighted graph, t>0	Sample-dependent	Full rank for exact heat kernel
Spectral repair	Finite raw Gram matrix	Finite repair	Recheck separation

**Table 3:** Status of the main AIT–kernel interfaces. “Conditional” means that the stated guarantee is established under the assumptions in the middle column; these conditions need not all be necessary.

Claim	Essential Dependency	Status
D2KE is PSD	Finite positive landmark measure	Proved
D2KE identifies laws	Positive-measure separation of every nonzero zero-mass signed measure	Exact criterion
$k_{\mathrm{Sol},U}\left(x,y\right){≍}_{U}2^{-K_{U}\left(\langle x,y\rangle \right)}$	Prefix-free halting domain and universal-semimeasure domination	Proved
Finite exact-*K* truncation converges	Omitted Kraft mass τ(F); the kernel and certification remain uncomputable	Proved, ideal
Finite program proxy is computable	Uniform conditional-feature error Δ	Conditional
MMD is profile distance	D2KE feature representation	Proved
The HSIC detects independence on countable spaces	Characteristic bounded marginal kernels	Proved
Expected AMI tracks Shannon MI	Finite computable source, with source description or auxiliary information	Conditional
The HSIC and MI are two-sided comparable	Fixed finite support, positive masses, and restricted product-kernel eigenvalue	Conditional
${\mathrm{KSD}}_{T_{P},k}\left(Q\right)={\mathrm{MMD}}_{k}\left(QT_{P},Q\right)$	Bounded PSD kernel and target-stationary operator	Proved
KSD detects Q=P	Characteristicity and uniqueness of the stationary law	Conditional
Named compressors approximate prefix complexity	Requires a separate compressor/source theorem	Not asserted

**Table 4:** Resolution of the actual D2KE Gram matrix with 24 uniform self-landmarks.

Length	Compressor	Distinct/276	Effective Rank	$\alpha_{k}$
64	gzip	33	1.073	$1.23\times 10^{-3}$
64	bz2	175	1.072	$6.23\times 10^{-4}$
256	gzip	66	1.148	$4.88\times 10^{-3}$
256	bz2	211	1.104	$2.41\times 10^{-3}$
1024	gzip	136	1.211	$9.87\times 10^{-3}$
1024	bz2	256	1.113	$3.48\times 10^{-3}$
4096	gzip	146	1.209	$8.38\times 10^{-3}$
4096	bz2	261	1.116	$3.94\times 10^{-3}$
16,384	gzip	131	1.201	$9.50\times 10^{-3}$
16,384	bz2	269	1.115	$3.47\times 10^{-3}$

**Table 5:** Rejection rates at α=0.05 with pointwise 95% Wilson intervals.

Method	Null Rejection	Alternative Rejection
D2KE/gzip	0.033 [0.013, 0.083]	1.000 [0.969, 1.000]
D2KE/bzip2	0.042 [0.018, 0.094]	1.000 [0.969, 1.000]
RBF/1-mers	0.067 [0.034, 0.126]	0.033 [0.013, 0.083]
RBF/2-mers	0.050 [0.023, 0.105]	0.058 [0.029, 0.116]
RBF/3-mers	0.033 [0.013, 0.083]	0.058 [0.029, 0.116]
RBF/4-mers	0.042 [0.018, 0.094]	0.058 [0.029, 0.116]
RBF/5-mers	0.058 [0.029, 0.116]	0.050 [0.023, 0.105]
RBF/6-mers	0.092 [0.052, 0.157]	0.800 [0.720, 0.862]

**Table 6:** Absolute error relative to the exact 240-landmark pool mean.

*m*	Mean Error	95th Percentile	Hoeffding Envelope
5	$9.46\times 10^{-3}$	$2.36\times 10^{-2}$	$6.07\times 10^{-1}$
10	$6.79\times 10^{-3}$	$1.57\times 10^{-2}$	$4.29\times 10^{-1}$
20	$4.65\times 10^{-3}$	$1.08\times 10^{-2}$	$3.04\times 10^{-1}$
40	$3.45\times 10^{-3}$	$8.23\times 10^{-3}$	$2.15\times 10^{-1}$
80	$2.31\times 10^{-3}$	$5.61\times 10^{-3}$	$1.52\times 10^{-1}$
160	$1.67\times 10^{-3}$	$3.88\times 10^{-3}$	$1.07\times 10^{-1}$

**Table 7:** Empirical KSD bins for 4000 candidate laws; these minima are over sampled candidates only.

${∥Q-P∥}_{1}$ Interval	Draws	Minimum KSD^2^	Median KSD^2^
[0.00,0.15)	4	$1.94\times 10^{-8}$	$3.74\times 10^{-7}$
[0.15,0.30)	203	$3.82\times 10^{-8}$	$1.47\times 10^{-6}$
[0.30,0.45)	708	$8.73\times 10^{-8}$	$4.10\times 10^{-6}$
[0.45,0.60)	739	$2.01\times 10^{-7}$	$8.77\times 10^{-6}$
[0.60,0.75)	656	$2.41\times 10^{-7}$	$1.20\times 10^{-5}$
[0.75,0.90)	617	$4.08\times 10^{-7}$	$1.81\times 10^{-5}$
[0.90,1.05)	517	$7.83\times 10^{-7}$	$2.87\times 10^{-5}$
[1.05,1.20)	319	$8.90\times 10^{-7}$	$3.86\times 10^{-5}$
[1.20,2.00]	237	$1.05\times 10^{-6}$	$8.00\times 10^{-5}$

**Table 8:** Reversal HSIC: an observed failure regime at α=0.05. These replicates are separate from the latent-type experiment.

ρ	Rejections/Trials	Rate [95% Wilson Interval]
0.00	6/60	0.100 [0.047, 0.201]
0.25	0/60	0.000 [0.000, 0.060]
0.50	2/60	0.033 [0.009, 0.114]
0.75	1/60	0.017 [0.003, 0.089]
1.00	2/60	0.033 [0.009, 0.114]

**Table 9:** Latent-type HSIC rejection at α=0.05.

ρ	Rejections/Trials	Rate [95% Wilson Interval]
0.00	2/50	0.040 [0.011, 0.135]
0.25	4/50	0.080 [0.032, 0.188]
0.50	13/50	0.260 [0.159, 0.396]
0.75	33/50	0.660 [0.522, 0.776]
1.00	39/50	0.780 [0.648, 0.872]

**Table 10:** Evidential scope of the corrected study. Layer B denotes finite D2KE identities; Layer C denotes compressor-dependent observations.

Study	Layer	Supported Conclusion
Profile and KSD routes	B	Numerical agreement of identities on saved finite inputs.
Resolution sweep	C	Separation on ten particular finite self-landmark supports.
Two-sample comparison	C	Behavior on a construction that preserves five local count orders.
Landmark and perturbation checks	B/C	Fixed-pool sampling behavior and finite profile error bounds.
KSD controls	B/C	Rank and stationary-law uniqueness have distinct roles.
HSIC demonstrations	C	Exploratory rejection rates for specified synthetic dependence.
Simplex boundary	B	A separating finite kernel may have a constant permutation statistic.

## Data Availability

All data in Section 8.10 are synthetic. The experimental configurations and summary results are reported in that section.
